# High-Resolution Underwater Mapping Using Side-Scan Sonar

**DOI:** 10.1371/journal.pone.0146396

**Published:** 2016-01-28

**Authors:** Antoni Burguera, Gabriel Oliver

**Affiliations:** Departament de Matemàtiques i Informàtica, Universitat de les Illes Balears, Palma de Mallorca, Spain; Dauphin Island Sea Lab, UNITED STATES

## Abstract

The goal of this study is to generate high-resolution sea floor maps using a *Side-Scan Sonar*(SSS). This is achieved by explicitly taking into account the SSS operation as follows. First, the raw sensor data is corrected by means of a physics-based SSS model. Second, the data is projected to the sea-floor. The errors involved in this projection are thoroughfully analysed. Third, a probabilistic SSS model is defined and used to estimate the probability of each sea-floor region to be observed. This probabilistic information is then used to weight the contribution of each SSS measurement to the map. Because of these models, arbitrary map resolutions can be achieved, even beyond the sensor resolution. Finally, a geometric map building method is presented and combined with the probabilistic approach. The resulting map is composed of two layers. The echo intensity layer holds the most likely echo intensities at each point in the sea-floor. The probabilistic layer contains information about how confident can the user or the higher control layers be about the echo intensity layer data. Experimental results have been conducted in a large subsea region.

## Introduction

### Overview

Acoustic sensors (*sonars*) are the modality of choice in underwater robotics [1–3]. One of these sensors is the *Side-Scan Sonar* (SSS) which provides echo intensity profiles of the sea bottom. Side-Scan Sonars are widely used in sea floor imagery, and should remain in the near future, basically for economic reasons, but also because of its ease of deployment: in some cases they have a towfish structure, so there is no need for complex mountings on *Autonomous Underwater Vehicles* (AUV), *Remotely Operated Vehicles* (ROV) or ships.

SSS is used for providing imagery to a wide variety of scientific applications in different underwater scenarios including deep sea, shallow waters, lakes or rivers.

Geological aspects like tectonic, volcanic or hydrothermal structures, as well as sedimented areas, among others, have been reported and studied in detail thanks to different sidescan sonar campaigns carried out in the last twenty years. Blondel in [4] presents a detailed compilation of representative examples using sidescan in such a scientific terrain.

The extension from geology to biology applications stems from the fact that many studies report a strong relation between benthic community structure and substrate type [5]. Thus, marine scientists have used sidescan sonar to assist in mapping and understanding the spatial extent of seabed habitats and benthic ecosystems.

Acoustic surveys typically cover tens or hundreds of square kilometers, thus conventional sidescan imagery interpretation made *by-eye* is hardly feasible and prone to subjectivity. As a consequence, different alternatives for automatic segmentation of benthic habitat using sidescan data have been proposed [6–8]. Comparing the output of the algorithms with the habitat ground truth from grab or video samplings indicates accuracies of the classification strategies above 80%

Still, the SSS imagery has some properties that jeopardize automated analysis [9]. For example, SSS provide misrepresented slices of the sea bottom [10], especially in non-flat terrains. Moreover, joining these slices to produce dense acoustic maps require accurate sensor pose data and significant interpolation in most cases. Additionally, SSS, as well as any other kind of sonar, ensonify the observed region unevenly [11] and, thus, the sensor itself influences the perception of the sea bottom.

The three aforementioned problems prevent, among others, the use of SSS data to build large scale sea-floor maps involving complex robot trajectories [12]. In most cases, the AUV is forced to move through straight transects to reduce the need for accurate localization and data interpolation. Even in these constrained scenarios some problems arise when trying to fuse data corresponding to overlapping regions.

A common approach to produce geometrically meaningful maps is the occupancy grid [13]. Although occupancy grids are aimed at distinguishing free and occupied regions, similar approaches have been applied with relative success in the context of SSS mapping [14, 15]. Unfortunately, the occupancy grid techniques still present some problems when used to construct dense SSS maps. These problems appear because most of the existing occupancy grid algorithms decompose the high-dimensional mapping problem into a set of one-dimensional problems, being the value of each cell estimated independently [16].

### Proposal

Our goal is to generate high-resolution maps, beyond the SSS resolution, of large sea floor regions using SSS by explicitly dealing with the aforementioned problems through the following four steps.

The first step, the *Swath Correction*, consists in improving the individual data items provided by the SSS. This is achieved in three stages. During the first one, the effects of the uneven ensonification pattern are removed using a physics-based sensor model [11]. Then, the central region of each data item (the *blind zone*) is detected and removed as it does not provide useful information. Afterwards, the data is projected to the actual sea floor. As, in most cases, bathymetry is not available, the floor is assumed to be flat to perform such projection. The error due to this assumption is properly modelled and evaluated, showing at which extent it is affordable.

The second step, *localization*, consists in estimating the AUV or the SSS pose. To this end, we use a *Doppler Velocity Log* (DVL) continuously and a *Global Positioning System* (GPS) when the AUV surfaces. The data provided by both sensors is fused using an *Extended Kalman Filter* (EKF). In order to deal with different sampling rates between the localization sensors and the SSS, a constant velocity model is adopted in the EKF prediction step.

During the third step, *Probabilistic Map Building*, a probabilistic map of the sea floor is built. This map does not hold information about the structure of the sea floor but about the probability of each mapped region to be observed by the SSS. To this end, some probabilistic SSS models as well as a method to combine the probabilistic information due to different overlapping measurements is used. These models share some similarities with the ones presented in [17] and used in [18, 19] with the following main differences. Our proposal focuses on fusing probabilistic and echo intensity information whereas the previous proposals were aimed at discarding useless data. Also, our proposal deals with echo intensity vectors whilst the previously mentioned require a single TOF reading with no echo intensity data.

During the last step, *Echo Intensity Map Building*, the probabilistic information is combined with the echo intensity information coming from the SSS to provide a meaningful representation of the sea floor. The proposed approach produces high resolution maps, even at higher resolutions than those of the SSS because it is able to combine the echo intensity information coming from several SSS measurements gathered at different poses whenever they overlap.

The better the resolution and the precision of the sidescan sonar is, the more accurate are the automatic classification algorithms results. Hence, the proposal here presented can benefit scientific applications that use sidescan sonar for mapping and segmentation.

**Fig 1:**
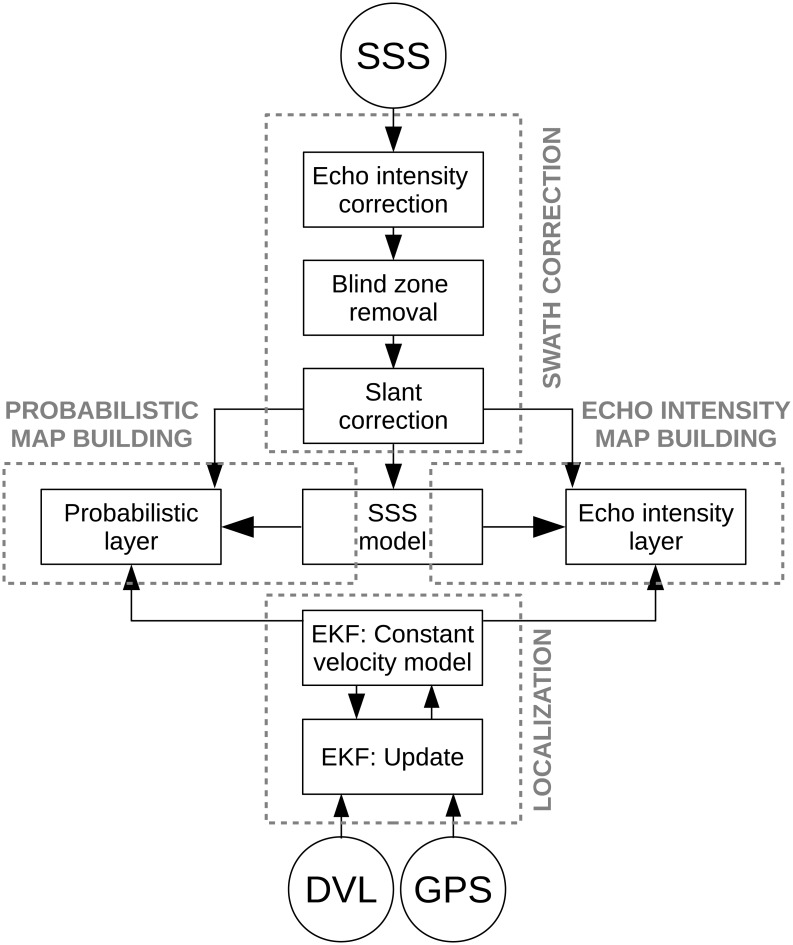
System overview. The main steps are *swath correction*, in charge of improving the individual SSS swaths; *probabilistic map building*, whose task is to generate a probability map estimating the likelihood of each pixel to be observed; *echo intensity map building*, which constructs a grid where each cell holds the most likely echo intensity corresponding to that cell; and *localization*, whose task is to estimate the AUV pose. These steps are not executed sequantially, but they are sensor-driven.

It is important to emphasize that these steps are not executed sequentially, but are sensor driven: when a new SSS measurement is gathered, it is improved and used to ammeliorate the probabilistic map and to extend the echo intensity map. When a new GPS or DVL measurement arrives, it is used to update the localization data. Fig 1 summarizes the aforementioned steps.

As a result of these steps, a map consisting of two layers is built. On the one hand, a probabilistic layer. The data in this layer is useful to, for example, help the user or the high-level control layers to decide which areas have been properly mapped and which ones deserve further re-observation. On the other hand, an echo intensity layer, which properly fuses all the obtained echo intensities provided by the SSS.

The novelties in this proposal are the following. First of all, the combination of the aforementioned techniques. For example, the proposed physics-based model to remove the uneven ensonification pattern has never been used in the context of dense acoustic map building. Also, the literature is scarce on models analysing the effects of assuming a flat floor. Thus, the proposed analysis is also one of the novelties in this paper.

Additionally, the proposed approach to build the echo intensity map deals with the problems of occupancy grid building by adopting a technique based on forward sensor models [16]. This approach explicitly takes into account the horizontal SSS opening, which is usually not considered in the literature. Finally, our proposal generates a two-layer map providing information not only about the actual sea floor structure but also about how confident the user or higher-level modules can be about that information.

Our proposal is tested using real SSS and DVL data gathered along a large subsea trajectory in *Port de Sóller* (Mallorca, Spain).

The paper is structured as follows. First, the SSS operation and the basic terminology is presented in Section The Side-Scan Sonar. Section The flat floor assumption models the effects of the flat floor assumption. All the aforementioned processes to improve the SSS data are detailed in Section Swath correction. Afterwards, Section Localization presents the specific EKF formulation used to deal with DVL and GPS sensors.

## The Side-Scan Sonar

### Overview

**Fig 2:**
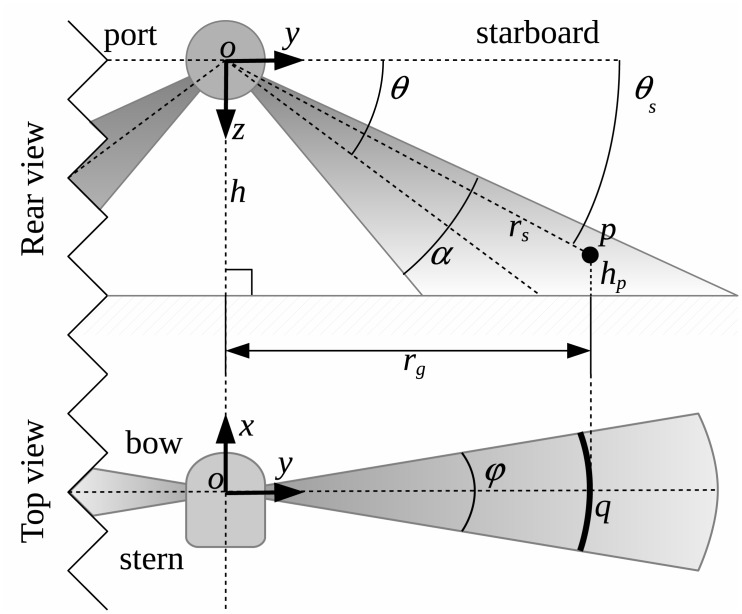
Side-scan sonar characterization. The AUV navigates at an altitude *h* with two SSS sensing heads symmetrically placed on port and starboard with a fixed angle *θ*. The angles *α* and *ϕ* are known as the *sensor openings* and model the sound expansion rate in the YZ and XY planes respectively. A point *p* in the ensonified region is usually expressed in polar coordinates using its *slant range* *r*_*s*_ and its *grazing angle* *θ*_*s*_, though sometimes the point altitude *h*_*p*_ is also used. Given this information, the point position can be expressed as a range *r*_*g*_ over the *Y* axis. Finally, due to the *ϕ* opening, the exact position of a point in the *XY* plane cannot be determined. Instead, the point can be anywhere over the arc *q*.

Fig 2 illustrates the operation of a SSS mounted on a AUV navigating at an altitude *h*. An AUV usually has two SSS heads, symmetrically placed on port and starboard with a fixed angle *θ*. Each sensing head periodically generates an ultrasonic pulse which, after reaching the sea floor, is partially scattered back to the sensor. The SSS then analyses the received echo to obtain information about the *ensonified region* (ER). The expansion of the sound wave with time is modelled by the sensor opening *α* in the YZ plane and the sensor opening *ϕ* in the XY plane.

The sensor operation is as follows. After each ultrasonic pulse emission, the sensor records the received echo intensities at fixed time intervals until a new pulse is emitted. Henceforth, this recorded data vector will be referred to as a *swath* and each of its items, which store an echo intensity providing information about the structure and reflectivity of the sea floor, will be referred to as a *bin*. The *Time Of Flight* (TOF) corresponding to each bin determines the *slant range* *r*_*s*_ of a point *p* in the ER. The sampling period determines the slant range resolution *δ*_*s*_ and the time between emitted pulses determines the maximum sensor range.

The acoustic echo intensity recorded in each bin is mainly influenced by the reflectivity of the sea floor, but also it is attenuated depending on the travelled distance and corrupted by the SSS ensonification pattern. Our proposal to reduce these undesired effects is referred to as the *intensity correction* [11] and is described in Section Intensity correction.

The position of a point *p* in the ER is usually expressed using the polar coordinates (*r*_*s*_, *θ*_*s*_) in the YZ plane. The angle *θ*_*s*_ is known as the *grazing angle* and can computed as follows:

$$\begin{array}{c} \theta_{s}=\arcsin\left(\frac{h-h_{p}}{r_{s}}\right) \end{array}$$ (1)

where *h*_*p*_ is the altitude of *p*. If *h*_*p*_ is unknown then *θ*_*s*_ cannot be computed, and the point responsible for the echo at *r*_*s*_ may be anywhere in the angular interval $\left[\theta -\frac{\alpha }{2},\theta +\frac{\alpha }{2}\right]$. Computing the elevation map of the points in the sea floor using only SSS data is a hard, time-consuming and error prone task addressed by few researchers [10]. Because of that, if no bathymetry is available through other sensors, a common approach is to assume that all the points in ER have zero altitude (*h*_*p*_ = 0). This assumption, which is known as the *flat floor assumption*, introduces an error when building maps. The effects of the flat floor assumption will be analysed in Section The flat floor assumption.

Because of the sensor opening *ϕ* a similar problem appears in the XY plane. The bin with slant range *r*_*s*_ is due to the objects lying in an arc *q*, as shown in Fig 2. One or more objects lying inside *q* may be responsible for the received echo intensity. This fact is usually neglected in the literature, assuming a pencil-like thin beam in the XY plane because *ϕ* is very small in most SSS. A novelty in this study is that the opening in the XY plane is not neglected, but properly used to fuse the information coming by overlapping ER.

The *ground range* *r*_*g*_ of a point *p* is the projection over the Y axis of the vector $\vec{op}$ joining the AUV reference frame and the point *p*. The ground range is useful to properly map each bin to its corresponding position in the sea floor. The process of computing the ground range for every bin is the so called *slant correction*. Our approach to this process is described in Section Slant correction.

### The swath structure

A typical SSS has two synchronized sensing heads and, thus, the swaths are provided in pairs: one provided by the port sensing head and one provided by the starboard sensing head. Let us, for the sake of clarity, join each two simultaneously gathered swaths in a single vector. Henceforth, the term *swath* will refer to this new vector.

**Fig 3:**
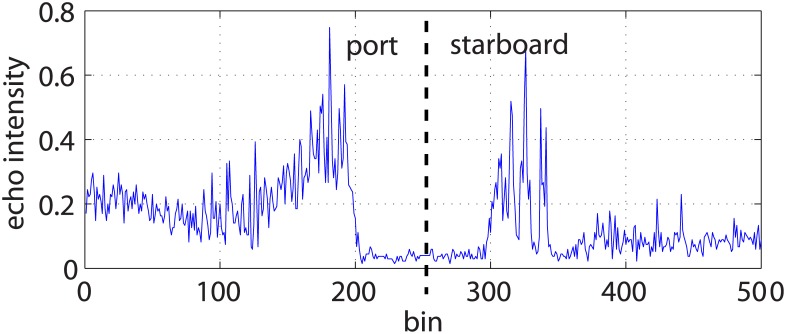
Single swath example. An example of a single swath corresponding to the port (bins 1 to 250) and starboard (bins 251 to 500) sensing heads of a particular SSS. The Y axis shows the obtained echo intensities normalized to values between 0 and 1. The central region with low echo intensities corresponds to the so-called *blind zone* and the first significant echo outside the blind zone is known as the *First Bottom Return* (FBR).

Fig 3 shows an example of a swath corresponding to the port (bins 1 to 250) and starboard (bins 251 to 500) sensing heads of a particular SSS. The central region with low echo intensities is the so called *blind zone* and it corresponds to those distances from the sensor where no sea floor was detected. Thus, echo intensity values in the blind zone are due to sensor noise and suspended particles in water. The first significant echo outside the blind zone is the *First Bottom Return* (FBR) and corresponds to the first sea floor point that produced an echo.

### Acoustic image formation

**Fig 4:**
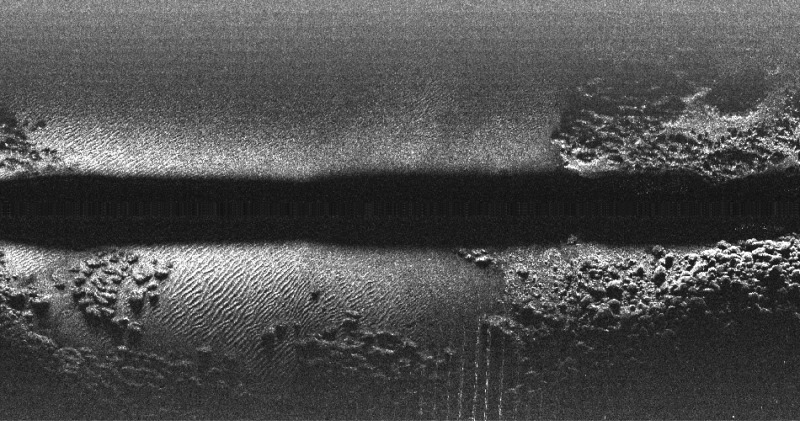
Acoustic image example. Example of acoustic image showing consecutively gathered swaths. Each column corresponds to a single swath and the echo intensities are mapped to a grayscale where black denotes no echo received and white represents the maximum echo intensity.

As the AUV moves, several swaths are gathered. By aggregating swaths, an *acoustic image* is built. The most basic form of acoustic image building consists in putting the swaths together, one next to the other. Fig 4 shows an example of this kind of images, where each echo intensity is mapped to a grayscale level so that lower intensities are darker. The changes in the black strip width, which is the blind zone, reflect changes in the AUV altitude: the higher the altitude, the wider the blind zone.

As the blind zone does not hold useful information about the environment, but noise and echoes due to suspended particles in water, it is desirable to detect and remove it. This process is known as *blind zone removal*. Our approach to the blind zone removal is described in Section Blind zone removal.

Although this approach may be sufficient for human inspection, it provides a distorted representation of the actual sea floor for two reasons. First, it does not take into account the specific AUV motion between swaths. Second, the vertical axis does not represent the ground range but the slant range. Solving the first problem involves AUV localization or SLAM techniques, as well as the use of interpolation and blending techniques to fill gaps and combine overlapping swaths. This is, precisely, the main goal of this paper and is going to be described in detail in further sections. As for the second problem, properly mapping slant ranges to the sea floor consists in performing the aforementioned slant correction.

## The flat floor assumption

### Overview

Properly projecting the SSS swaths to the sea floor is an error prone, complex and time consuming task [10]. Thus, unless bathymetry is available, it is common to assume that all the objects in the ER have zero altitude (*h*_*p*_ = 0) with respect to the point where the AUV altitude is measured. This is the so called flat floor assumption.

At the extent of the authors knowledge, very few studies exist that explicitly analyse the errors introduced by the flat floor assumption in SSS imagery. For example, [20] states the problem but focuses on correcting its effects using bathymetry. [21] also states the problems but mainly accounts for them when analysing acoustic images, neglecting their effects during the acoustic image formation. In general it is assumed that if the terrain roughness is small when compared to the AUV altitude, the errors are neglectable. This is the case of [2] or [11]. In these studies, the ocean floor is assumed to be locally flat and experimentally shown that this assumption is affordable in real underwater scenarios with relatively flat floors.

The goal of this Section is to model the errors due to the flat floor assumption in order to quantify them and show with which kind of sensors and environments such assumption is reasonable.

### Error model

**Fig 5:**
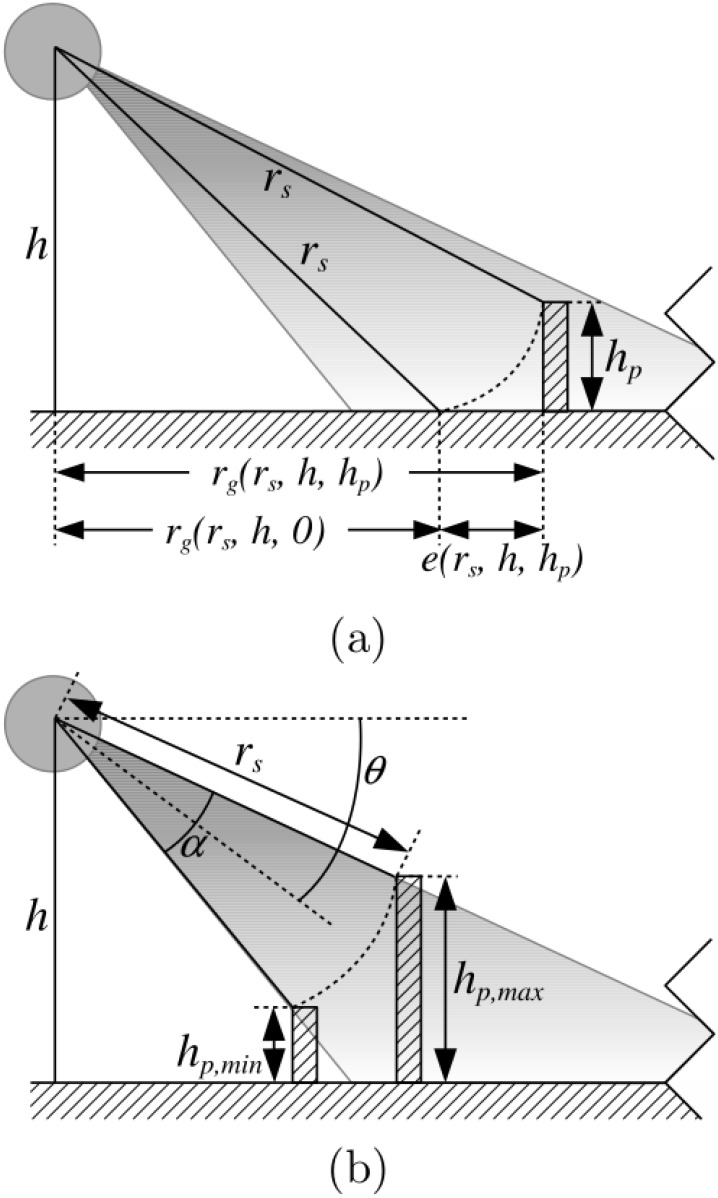
The problems of the flat floor assumption. (a) Given a measured slant range *r*_*s*_ and AUV altitude *h*, if the height *h*_*p*_ of the object responsible for the echo is unknown and assumed to be zero following the flat floor assumption, an error *e* appears in the estimated ground range *r*_*g*_. This error is defined as the difference between the actual ground range and the one obtained if the detected object is assumed to have zero height. (b) Given a slant range *r*_*s*_ and knowing the angular sensor placement *θ* and opening *α* the true object height must be in the interval [*h*_*p*, *min*_, *h*_*p*, *max*_].

From Figs 2 and 5a it is easy to see that the ground range *r*_*g*_ can be expressed as a function of the measured slant range *r*_*s*_, the AUV altitude *h* and the altitude *h*_*p*_ of the detected object as follows:

$$\begin{array}{c} r_{g}\left(r_{s},h,h_{p}\right)=\sqrt{r_{s}^{2}-{\left(h-h_{p}\right)}^{2}} \end{array}$$ (2)

Performing the flat floor assumption means using *h*_*p*_ = 0. Accordingly, we define the flat floor assumption error *e* (see Fig 5a) as the difference between the true ground range and the one corresponding to *h*_*p*_ = 0 as follows:

$$\begin{array}{c} e\left(r_{s},h,h_{p}\right)=\left|r_{g}\left(r_{s},h,h_{p}\right)-r_{g}\left(r_{s},h,0\right)\right| \end{array}$$ (3)

The slant range *r*_*s*_ is computed from the TOF of each bin in the swath and the altitude can be measured by sensors commonly available in underwater robots, such as DVL or the SSS itself. Moreover, in case no sensor measuring *h* is available, it can be easily computed from the FBR, as it will be shown later. To the contrary, *h*_*p*_ is unknown. Thus, for a given bin and time, the error only depends on the unknown object altitude.

We define an error to be *fully neglectable* if it is below the SSS resolution. That is, we assume that errors below that resolution will not affect the acoustic image formation.

### Minimum and maximum object altitudes

Given an altitude *h* and a slant range *r*_*s*_, not all object altitudes *h*_*p*_ are possible, especially if the opening *α* and the angle *θ* are considered. From Figs 2 and 5b the minimum and maximum possible object altitudes are as follows:

$$\begin{array}{ccc} h_{p,min}\left(r_{s},h\right) & = & h-r_{s}\cdot \sin\left(\theta +\frac{\alpha }{2}\right) \end{array}$$ (4)

$$\begin{array}{ccc} h_{p,max}\left(r_{s},h\right) & = & h-r_{s}\cdot \sin\left(\theta -\frac{\alpha }{2}\right) \end{array}$$ (5)

if $\theta -\frac{\alpha }{2}\geq 0$ and $\theta +\frac{\alpha }{2}\leq \frac{\pi }{2}$. These two conditions mean that the SSS is mounted on the AUV so that the acoustic beam never crosses the *Y* or the *Z* axes, which happens in most SSS configurations. Let the minimum and maximum object altitudes leading to fully neglectable errors be denoted as $h_{p,min}^{\prime }\left(r_{s},h\right)$ and $h_{p,max}^{\prime }\left(r_{s},h\right)$ respectively.

### Across-track slope

There is a final consideration to be performed. Even if we assume that protuberances and holes in the sea floor are within the fully neglectable error ranges, the ocean floor slope in the across-track direction (Y axis) has also to be taken into account, as it may lead to significant changes in *h*_*p*_. The minimum and maximum terrain slopes leading to fully neglectable errors can be computed as follows:

$$\begin{array}{ccc} s_{min}\left(h\right) & = & 100\cdot \frac{h_{p,min}^{\prime }\left(r_{s,max},h\right)}{r_{g}\left(r_{s,max},h,h_{p,min}^{\prime }\right)} \end{array}$$ (6)

$$\begin{array}{ccc} s_{max}\left(h\right) & = & 100\cdot \frac{h_{p,max}^{\prime }\left(r_{s,max},h\right)}{r_{g}\left(r_{s,max},h,h_{p,max}^{\prime }\right)} \end{array}$$ (7)

where *r*_*s*, *max*_ is the sensor range.

It should be noticed that slopes outside this range lead to not fully neglectable errors when considering the absolute ground range, but the errors would be much smaller locally, leading to almost locally perfect acoustic images for a much wider range of slopes.

As a conclusion, the models presented in this Section make it possible to quantify the errors due to the flat floor assumption. These models will be configured with our particular sensor parameters in Section Experimantal result, showing that the flat floor assumption is more than reasonable with our specific AUV configuration and also affordable in similar missions. For this reason, the flat floor assumption is going to be performed throughout this paper.

## Swath correction

### Intensity correction

It can be observed in Figs 3 and 4 that the echo intensities are much higher in the regions surrounding the blind zone. This effect is due to a non homogeneous ensonification leading to brightness variations in the resulting acoustic image that may difficult further automated processing. Some studies deal with this problem, but mainly using some environment dependant heuristics [9].

Our proposal has a well founded theoretical basis [11], as it relies on sea bed reflectivity and sound propagation models. To remove the variable ensonification component from the acoustic image, we model the sea floor as a Lambertian surface [10, 22], which scatters incident energy uniformly in all directions. Under this assumption, the echo intensity *I*^*m*^(*p*) returned by a SSS measurement *m* from a sea floor point *p* = (*r*_*s*_, *θ*_*s*_) is modelled as follows:

$$\begin{array}{c} I^{m}\left(p\right)=K\cdot \phi \left(p\right)\cdot R^{m}\left(p\right)\cdot \cos\left(\beta^{m}\left(p\right)\right) \end{array}$$ (8)

where *ϕ*(*p*) denotes the ensonification intensity, *R*^*m*^(*p*) is the reflectivity of the sea floor, *β*^*m*^(*p*) is the incidence angle, which coincides with the grazing angle *θ*_*s*_ under the flat floor assumption, and *K* is a normalization constant.

We derived the ensonification intensity model from the *sensitivity pattern* model proposed by Kleeman and Kuc [23]. Accordingly, *ϕ* can be expressed as follows:

$$\begin{array}{c} \phi \left(r_{s},\theta_{s}\right)=\frac{Mfa^{4}}{r_{s}^{2}}{\left(\frac{2J_{1}\left(\frac{2\pi }{\lambda }a\sin\left(\theta -\theta_{s}\right)\right)}{\frac{2\pi }{\lambda }a\sin\left(\theta -\theta_{s}\right)}\right)}^{2} \end{array}$$ (9)

where *M* is a proportionality constant, *J*_1_ is the Bessel function of the first kind of order 1, *λ* and *f* are the emitted pulse wavelength and frequency respectively and *a* is the transducer radius. This Equation accounts for the two main aspects that modify the intensity perceived at a given point in the sea floor: its angular position with respect to the sensor acoustic axis and its distance to the sensor origin, which is related to the sound attenuation with the travelled distance.

As it may be difficult to find an accurate value for the transducer radius *a* from the sensor specs, we propose the following procedure. Let *θ*_0_ be the first occurring *θ*_*s*_ producing *ϕ*(.) = 0. Thus, *θ*_0_ defines the main sonar lobe: the ensonification intensities corresponding to grazing angles in the interval [*θ*_0_, 2*θ* − *θ*_0_] are due to the main sonar lobe, whilst those outside the interval are produced by the secondary lobes. The value of *θ*_0_ can be derived [23] from Eq 9 and is as follows:

$$\begin{array}{c} \theta_{0}=\theta -\arcsin\left(\frac{0.61\lambda }{a}\right) \end{array}$$ (10)

Let us assume that the whole sensor opening *α* corresponds to the main lobe, which is reasonable because manufacturers usually build sensors in this way. The grazing angle corresponding to the positive boundary of the ensonified region according to the opening is *θ* − *α*/2 (See Fig 2). Thus, using *θ*_0_ = *θ* − *α*/2 in Eq 10 leads to a fair approximation of the transducer radius *a*:

$$\begin{array}{c} a=\frac{0.61\lambda }{\sin\left(\frac{\alpha }{2}\right)} \end{array}$$ (11)

According to Eq 8, the measured echo intensities, that is, the bin values in each swath, depend on the sea floor reflectivity, the ensonification intensity and the incidence angle. An acoustic image taking only into account the sea floor reflectivity would be a more realistic representation of the environment, as it discards the effects due to the sensor itself. As we have an ensonification model and we know the incidence angles, we can express the corrected value for each point *p* in the ER as follows:

$$\begin{array}{c} R^{m}\left(p\right)=K^{\prime }\cdot \frac{I^{m}\left(p\right)}{\phi \left(p\right)\cdot \cos(\beta^{m}\left(p\right))} \end{array}$$ (12)

where *K*′ = (*K* ⋅ *M*)^−1^. Thus, the acoustic image built from *R*^*m*^(*p*) constitutes the intensity corrected image of *I*^*m*^(*p*).

Further methods described in this paper make use of the corrected swath *R*^*m*^(*p*). However, all of them can also use the echo intensities *I*^*m*^(*p*). Because of that, to ease notation, the terms *I*^*m*^(*p*) and echo intensity will be used to refer to a swath and the meaning of the values it holds respectively, either if they are actually echo intensities or the corresponding reflectivity values.

### Slant correction

As stated previously, the process of projecting each bin to its corresponding position in the sea floor is known as slant correction. Under the flat floor assumption, this can be easily achieved through Eq 2 using *h*_*p*_ = 0. However, from an algorithmic point of view and to avoid gaps in the slant corrected swath, it is preferable to to have the slant range as a function of the ground range:

$$\begin{array}{c} r_{s}\left(r_{g},h,h_{p}\right)=\sqrt{r_{g}^{2}+{\left(h-h_{p}\right)}^{2}} \end{array}$$ (13)

In this way, for every possible ground range, the slant range can be computed and the corresponding echo intensity value obtained. The ground range resolution *δ*_*g*_ when performing the slant correction can be chosen depending on the desired granularity. However, a good choice is to use the same value as the slant range resolution *δ*_*s*_, which is sensor dependant, and that is the approach adopted in this paper. Also, as the slant range corresponding to a specific ground range may lie between two bins, linear interpolation is used to obtain the echo intensity. Algorithm 1 summarizes this process. The term *I*^*m*^(⋅) denotes the input swath. It is a vector where each value represents the echo intensity corresponding to a single bin. The term $I_{g}^{m}\left(\cdot \right)$ denotes the corrected swath. It is a vector where each value represents the echo intensity corresponding to a bin in the ground plane. The size of a bin in the ground plane is the ground range resolution *δ*_*g*_.

**Algorithm 1:** Slant correction.

**Data**: *I*^*m*^(⋅): Input swath. Vector containing the echo intensity for each bin.
**Result**: $I_{g}^{m}\left(\cdot \right)$: Corrected swath. Vector containing the echo intensity for each bin.
1 *r*_*g*, *min*_ = *r*_*g*_(*r*_*s*, *min*_, *h*, 0);
2 *r*_*g*, *max*_ = *r*_*g*_(*r*_*s*, *max*_, *h*, 0);
3 *δ*_*g*_ ← *δ*_*s*_;
4 **for** *r*_*g*_ ← *r*_*g*, *min*_ ***to*** *r*_*g*, *max*_ ***step*** *δ*_*g*_ **do**
5 $r\leftarrow \frac{r_{s}\left(r_{g},h,0\right)}{\delta_{s}}$;
6 *w*_1_ ← *r* − ⌊*r*⌋;
7 *w*_2_ ← 1 − *w*_1_;
8 $I_{g}^{m}\left(\left|\frac{r_{g}}{\delta_{g}}\right|\right)\leftarrow w_{2}\cdot I^{m}\left(\left\lfloor r\right\rfloor \right)+w_{1}\cdot I^{m}\left(\left\lceil r\right\rceil \right)$;
9 **end**

For the sake of simplicity, henceforth the term *swath* will refer to the ground projected swath $I_{g}^{m}\left(\cdot \right)$ unless the contrary is stated.

### Blind zone removal

Under the flat floor assumption, the AUV altitude *h* and the ground range corresponding to the FBR (*r*_*FBR*_) are related through *θ* and the sensor opening *α* as follows:

$$\begin{array}{c} r_{FBR}=\frac{h}{\tan\left(\theta +\frac{\alpha }{2}\right)} \end{array}$$ (14)

If the altitude *h* is known at each time step, the *r*_*FBR*_ can be computed and used to perform the blind zone removal. Assuming that the two SSS sensing heads are symmetrically placed on port and starboard, the blind zone removal consists in discarding all the bins whose ground ranges lie between −*r*_*FBR*_ and *r*_*FBR*_. If they are not symmetrically placed, two *r*_*FBR*_ should be computed, one for each sensing head. As stated previously, these bins do not hold useful information about the environment, but noise or echoes from suspended particles in water and thus they shall be discarded.

It should also be noticed that, in the unlikely case that the altitude *h* is not available, the FBR could be determined using some signal processing technique and the altitude *h* computed using Eq 14.

## Localization

### Overview

From the localization perspective, the roll and pitch angles are assumed to be zero, which is reasonable in most surveying missions where the robot control is designed to keep these angles to that value. Thus, the localization problem is a 4DOF problem where *x*, *y*, *z* and the yaw angle *ψ* have to be estimated.

Without loss of generality, this Section focuses on the localization sensors used in our particular configuration: a DVL providing velocities over the three axes and a GPS providing absolute pose when the AUV emerges. Additionally, our DVL provides altitude information and, also, absolute heading by means of its internal compass. The proposed localization approach relies on the standard EKF formulation, similarly to [14]. Accordingly, other sensor configurations could be easily used.

It has to be taken into account that localization, in this study, is not the goal but only the mean to produce accurate sea floor maps using SSS. Thus, the localization modules also have to pay attention to the different sampling rates between the localization sensors and the SSS. In our particular case, SSS provides data at higher rate than DVL and GPS. Thus, the proposed localization approach has to be able to provide the best possible pose estimate even when no DVL or GPS is available. To this end, DVL and GPS data are used in the EKF update step and a constant velocity model is used in the prediction step.

### The constant velocity model

The state vector is composed of the 3D AUV position and the yaw angle and the corresponding velocities, which are mandatory because of the adopted constant velocity model and also to allow using the DVL data during the update step:

$$\begin{array}{c} X_{t}={\left[x_{t},y_{t},z_{t},\psi_{t},{\dot{x}}_{t},{\dot{y}}_{t},{\dot{z}}_{t},{\dot{\psi }}_{t}\right]}^{T} \end{array}$$ (15)

According to the constant velocity model, the prediction is performed as follows:

$$\begin{array}{c} X_{t}=\left[\begin{array}{c} x_{t-1}+\left({\dot{x}}_{t-1}c_{t-1}-{\dot{y}}_{t-1}s_{t-1}\right)\Delta T\\ y_{t-1}+\left({\dot{x}}_{t-1}s_{t-1}+{\dot{y}}_{t-1}c_{t-1}\right)\Delta T\\ z_{t-1}+{\dot{z}}_{t-1}\Delta T\\ \psi_{t-1}+{\dot{\psi }}_{t-1}\Delta T\\ {\dot{x}}_{t-1}\\ {\dot{y}}_{t-1}\\ {\dot{z}}_{t-1}\\ {\dot{\psi }}_{t-1} \end{array}\right] \end{array}$$ (16)

where *s*_*t* − 1_ and *c*_*t* − 1_ denote sin(*ψ*_*t* − 1_) and cos(*ψ*_*t*−1_) respectively. By means of this model, when no sensor data is available, the prediction step will assume that velocities are the same as the last time they were measured and provide a pose estimate accordingly.

### The update step

The update step has to take into account the two aforementioned sensors (DVL and GPS), that may provide data at different sampling rates. As a matter of fact, GPS updates are likely to be very infrequent as they only happen when the AUV surfaces. As for used DVL device, it provides altitude, heading and velocities as follows:

$$\begin{array}{c} z_{DVL}={\left[z_{DVL},\psi_{DVL},{\dot{x}}_{DVL},{\dot{y}}_{DVL},{\dot{z}}_{DVL},{\dot{\psi }}_{DVL}\right]}^{T} \end{array}$$ (17)

The GPS data, when available, is adapted to the following format:

$$\begin{array}{c} z_{GPS}={\left[x_{GPS},y_{GPS},z_{GPS}\right]}^{T} \end{array}$$ (18)

Also, when the mission starts, the GPS position and the absolute heading are used to initialize the state vector.

As these sensors provide data directly related to specific state vector items, the measurement functions *h*_*DVL*_ and *h*_*GPS*_ and associated Jacobian matrices *H*_*DVL*_ and *H*_*GPS*_ are straightforward:

$$\begin{array}{ccc} h_{DVL} & = & {\left[z_{t},\psi_{t},{\dot{x}}_{t},{\dot{y}}_{t},{\dot{z}}_{t},{\dot{\psi }}_{t}\right]}^{T} \end{array}$$ (19)

$$\begin{array}{ccc} H_{DVL} & = & \left[\begin{array}{cccccccc} 0 & 0 & 1 & 0 & 0 & 0 & 0 & 0\\ 0 & 0 & 0 & 1 & 0 & 0 & 0 & 0\\ 0 & 0 & 0 & 0 & 1 & 0 & 0 & 0\\ 0 & 0 & 0 & 0 & 0 & 1 & 0 & 0\\ 0 & 0 & 0 & 0 & 0 & 0 & 1 & 0\\ 0 & 0 & 0 & 0 & 0 & 0 & 0 & 1 \end{array}\right] \end{array}$$ (20)

$$\begin{array}{ccc} h_{GPS} & = & {\left[x_{t},y_{t},z_{t}\right]}^{T} \end{array}$$ (21)

$$\begin{array}{ccc} H_{GPS} & = & \left[\begin{array}{ccc} 1 & 0 & 0\\ 0 & 1 & 0\\ 0 & 0 & 1 \end{array}\right] \end{array}$$ (22)

Localization is achieved by using the constant velocity model in the EKF prediction step and the aforedescribed measurement vectors and functions in the update step.

## Probabilistic map building

### Overview

At this point, we have described how each individual swath can be improved after the SSS gathers it and also how to properly estimate the AUV pose even at higher frequencies than those of the localization sensors. Also, we have shown the extent at which the flat floor assumption is affordable and, as the experimental results will corroborate, that it is reasonable to perform such assumption in a wide range of scenarios including ours.

This Section describes how a high resolution and high quality map of the subsea floor can be built using the improved swaths. This map is a 2D representation of the sea floor and, thus, it focuses on the *XY* plane (Fig 2). Being our proposal based on probability theory, we first describe the probabilistic SSS model and the probability map and, then, a method to combine the probabilistic information with the swath data to build the map.

### The SSS model

In the context of this paper, a map *M* is composed of two layers: the probability map *M*_*P*_ and the echo intensity map *M*_*I*_. Both are matrices of *R* rows and *C* columns where each cell represents a squared area of the sea floor of *δ*_*M*_ × *δ*_*M*_ meters. For the sake of clarity, let the cells be referred to as *pixels* and *δ*_*M*_ as the *map resolution*. In the case of *M*_*P*_ each pixel holds information about its own probability to be observed. As for *M*_*I*_ each pixel holds information about the echo intensity of the sea floor area it represents. We would like to emphasize that, as stated previously, the term echo intensity may refer to the raw echo intensity or to the corrected one processed as described in Section Intensity correction.

Let us express a pixel *q* by the coordinates of its four corners *q*_0_, *q*_1_, *q*_2_ and *q*_3_. Let $q_{i}^{m}=\left(r_{q,i}^{m},\theta_{q,i}^{m}\right)$ be the polar coordinates of the corner *q*_*i*_ in the ground plane with respect to the reference frame of a specific SSS measurement *m* ∈ *S*, where *S* denotes the set of all the gathered measurements, projected to the ground plane. In order to compute the position of the map pixel corners relative to the the SSS measurement, the position of the SSS measurement with respect to the map is required. This position is provided by the localization modules and, as described in Section Localization, it is available at the desired frequency thanks to the adopted constant velocity model.

**Fig 6:**
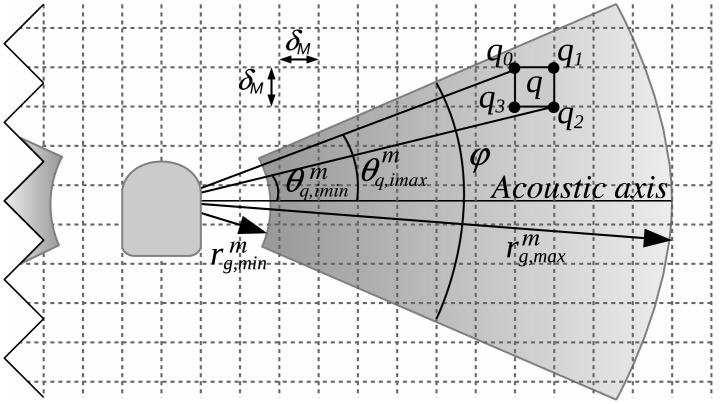
The SSS model. Each map cell or pixel represents a squared area of *δ*_*M*_ × *δ*_*M*_ meters. Each cell can be represented by the coordinates *q*_0_, *q*_1_, *q*_2_, *q*_3_ of its four vertexes. The terms $\theta_{q,imin}^{m}$ and $\theta_{q,imax}^{m}$ denote the angle with respect to the AUV reference frame of the rightmost and leftmost vertexes of a particular cell as observed from the AUV. The terms $r_{g,min}^{m}$ and $r_{g,max}^{m}$ denote the minimum and maximum ground range, respectively, that the SSS can reach.

Let *imin* and *imax* be the indexes of two pixels corners so that $\theta_{q,imin}^{m}$ and $\theta_{q,imax}^{m}$ are the rightmost and the leftmost angles. Finally, let $r_{g,min}^{m}$ and $r_{g,max}^{m}$ denote the minimum and maximum ground range, respectively, that the SSS can reach. Fig 6 illustrates these concepts.

The first step is to model the *Probability Density Function* (PDF) *p* of a point *q*_*i*_ to be observed by the SSS measurement. A realistic, physics-based, approach is to adapt Eq 9 to model the probabilities so that the points in the ER receiving higher ensonification energy are more likely to be observed. However, this approach is time consuming and the computation requirements when building large maps may be extremely large. Instead, our proposal is to model the probabilities as a Gaussian whose parameter is the angle $\theta_{q,i}^{m}$, which captures the shape of the acoustic main lobe. Accordingly, the PDF $p(\theta_{q,i}^{m})$ is defined as follows if the point range is within the observable range:

$$\begin{array}{c} p\left(\theta_{q,i}^{m}\right)=\frac{2}{\varphi \sqrt{2\pi }}e^{\left(\frac{-{\left(\theta_{q,i}^{m}\right)}^{2}}{2{\left(\frac{\varphi }{2}\right)}^{2}}\right)} \end{array}$$ (23)

In this way the probability is maximum when the point lies on the acoustic axis and decreases as the angular position gets farther from the acoustic axis. The standard deviation is set to $\frac{\varphi }{2}$ so that the 95% of the probability lies in the sonar main acoustic lobe.

The next step is to compute the probability of a pixel *q* to be observed by the measurement *m*. In other words, the goal is to compute the probability of a squared region of the sea floor to be observed by *m*. Taking into account that the PDF in Eq 23 only depends on the angle $\theta_{q,i}^{m}$ and that a pixel *q* lies in the angular interval [$\theta_{q,imin}^{m}$, $\theta_{q,imax}^{m}$], the probability $P_{G}^{m}\left(q\right)$ can be computed as follows:

$$\begin{array}{c} P_{G}^{m}\left(q\right)=\int_{\theta_{q,imin}^{m}}^{\theta_{q,imax}^{m}}p(\theta )d\theta \end{array}$$ (24)

As this Equation refers to a whole pixel, it may happen that the pixel partially lies within the observable range. Our proposal in this case is to compute the probability according to Eq 24 if the pixel is either fully or partially inside the observable range and to assume the probability is zero otherwise.

Unfortunately, using Eq 24 may be time consuming. It has to be taken into account that for every pixel in the map the model has to be computed for each gathered measurement, as it will be shown later. In order to reduce the computational cost, two additional models are proposed.

The first additional model consists in using a triangular PDF so that the probability is maximum when $\theta_{q,i}^{m}=0$ and zero outside the angular interval [$-\frac{\varphi }{2}$, $\frac{\varphi }{2}$], decreasing linearly when approaching the interval boundaries. By integrating this PDF similarly to Eq 24, the probability of a pixel *q* within the measurement range to be observed is:

$$\begin{array}{c} P_{T}^{m}\left(q\right)=\left\{\begin{array}{c} \frac{-2\left(\theta_{0}-\theta_{1}\right)\cdot \left(\theta_{0}+\theta_{1}-2b_{0}\right)}{{\left(b_{0}-b_{1}\right)}^{2}},\theta_{1}\leq 0\\ \frac{2\left(\theta_{0}-\theta_{1}\right)\cdot \left(\theta_{0}+\theta_{1}-2b_{0}\right)}{{\left(b_{0}-b_{1}\right)}^{2}},\theta_{0}\geq 0\\ \frac{4\theta_{0}b_{0}+b_{1}\left(4\theta_{1}-4b_{0}\right)-2\left(\theta_{0}^{2}+\theta_{1}^{2}\right))}{{\left(b0-b1\right)}^{2}}-1,otherwise \end{array}\right. \end{array}$$ (25)

where $b_{0}=-\frac{\varphi }{2}$, $b_{1}=\frac{\varphi }{2}$ and $\theta_{0}=max\left(\theta_{q,imin}^{m},-\frac{\varphi }{2}\right)<\theta_{1}=min\left(\theta_{q,imax}^{m},\frac{\varphi }{2}\right)$.

The second additional model aimed at reducing the computational cost consists in using a uniform PDF whose variable is $\theta_{q,i}^{m}$. Accordingly, the probability of a pixel *q* within the measurement range to be observed is:

$$\begin{array}{c} P_{U}^{m}\left(q\right)=\frac{\theta_{1}-\theta_{0}}{\varphi } \end{array}$$ (26)

In this Section three approaches to model the probability of a map cell *q* to be observed by a particular measurement *m* have been proposed. Henceforward, these three approaches will be referred to as the *Gaussian Approach*, the *Triangular Approach* and the *Uniform Approach* and their associated probabilities as $P_{G}^{m}$, $P_{T}^{m}$ and $P_{U}^{m}$ respectively. Also, as they can be used interchangeably, depending on the desired accuracy and available computational resources, the notation *P*^*m*^ will be used to refer to any of the three approaches to compute the probabilities. Some examples involving the three approaches are shown in Section Experimental results.

### The probability layer

This Section focuses on building the probability layer *M*_*P*_ of the map. Given the set of SSS measurements *m* ∈ *S*, a pixel *q* ∈ *M*_*P*_ may not have been observed by any measurement or it may have been observed by one or more measurements.

**Fig 7:**
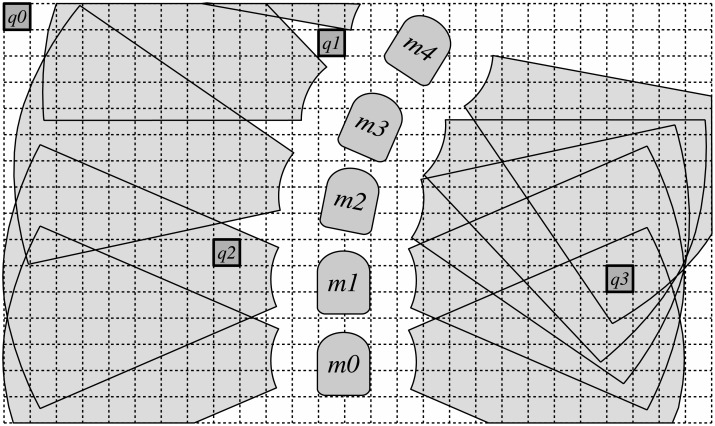
Possible pixel situations. A pixel may not be observed because it lies outside the maximum measurement range (*q*0) or because, due to the AUV motion, it lies between two observed regions (*q*1). A pixel may be observed by a single measurement (*q*2) or by several measurements (*q*3).

Fig 7 illustrates the four situations in which a pixel can be. Pixels *q*0 and *q*1 have not been observed by any measurement and, thus, their probability to be observed is zero. However, when building the reflectivity map these two pixels have to be considered differently, as it will be shown in Section Geometric map building.

Pixel *q*2 may have been observed by *m*1 with probability *P*^*m*1^(*q*2), as described in Section The SSS model. As for pixel *q*3 it may have been observed by *m*0, *m*1, *m*2, *m*3 or *m*4 with probabilities *P*^*m*0^(*q*3), *P*^*m*1^(*q*3), *P*^*m*2^(*q*3), *P*^*m*3^(*q*3) and *P*^*m*4^(*q*3) respectively. Accordingly, the probability of *q*3 to be observed is:

$$\begin{array}{c} P\left(q3\right)=P\left(E_{q3}^{m0}\cup E_{q3}^{m1}\cup E_{q3}^{m2}\cup E_{q3}^{m3}\cup E_{q3}^{m4}\right) \end{array}$$ (27)

where $E_{qj}^{mi}$ denotes the event *pixel qj observed by measurement mi* and has probability *P*^*mi*^(*qj*).

In general,

$$\begin{array}{c} P\left(q\right)=P\left(\underset{m\in S_{q}}{\cup }E_{q}^{m}\right) \end{array}$$ (28)

where *S*_*q*_ ⊂ *S* is the set of measurements that may have observed *q*. That is,

$$\begin{array}{c} S_{q}=\left\{m\in S|P^{m}\left(q\right)\geq \varepsilon \right\} \end{array}$$ (29)

As for *ε*, its value depends on the SSS model. If the Triangular or the Uniform models are used, then *ε* = 0. In the case of the Gaussian model *ε* should be greater than zero. Alternatively, a geometric approach could be used by including in *S*_*q*_ the set of measurements whose observed sector, defined by $r_{g,min}^{m}$, $r_{g,max}^{m}$ and *ϕ*, does contain *q*.

Assuming that the events $E_{qj}^{mi}$ are mutually independent but not mutually exclusive, the probability *P*(*q*) can be computed as:

$$\begin{array}{c} P\left(q\right)=P{\left(\underset{m\in S_{q}}{\cap }{\left(E_{q}^{m}\right)}^{C}\right)}^{C}=1-\prod_{m\in S_{q}}\left(1-P^{m}\left(q\right)\right) \end{array}$$ (30)

Algorithm 2 summarizes the process to build the probabilistic layer. For each pixel, the coordinates *q*_*G*_ of its four corners with respect to a global reference frame are computed in line 3. Then, the set *S*_*q*_ of measurements that may observe the current pixel is computed as follows. For each measurement *m*, its global coordinates are requested to the localization modules in line 7. Then, a fast check is performed in line 8: if the measurement does not intersect with the map rectangle, it is discarded without further tests. This is crucial to reduce the execution time. Otherwise, line 9 computes the polar coordinates of the four corners of the current pixel with respect to the current measurement as well as the rightmost and leftmost angles ($\theta_{q,imin}^{m}$ and $\theta_{q,imax}^{m}$). Then, line 10 decides if the measurement *m* may observe the pixel *q* using either Eq 29 or a geometric criteria. If it may be observed, both *m* and *q* are stored in *S*_*q*_ and *Q* respectively.

**Algorithm 2:** Probabilistic layer building.

**Input**:	*R*, *C*:	Map dimensions
	*δ*_*M*_:	Map resolution
	*M*_*x*0_, *M*_*y*0_:	Global coordinates of the map origin
	*S*:	Set of SSS measurements
**Result**: *M*_*p*_: Probabilistic layer.		
1 **for** *row* = 1 **to** *R* **do**		
2 **for** *col* = 1 **to** *C* **do**		
3 *q*_*G*_ ← *cell*_*to*_*global*(*row*, *col*, *δ*_*M*_, *M*_*x*0_, *M*_*y*0_);		
4 *S*_*q*_ ← ⌀;		
5 *Q* ← ⌀;		
// Build the set of measurements that may observe the current map cell.		
6 **foreach** *m* ∈ *S* **do**		
7 *m*_*G*_ ← *localization*(*m*);		
8 **if** *in_map*(*m*_*G*_, *M*_*x*0_, *M*_*y*0_, *R*, *C*, *δ*_*M*_) **then**		
9 *q* ← cell_to_local(*q*_*G*_, *m*_*G*_);		
10 **if** observed(*q*, *m*) **then**		
11 *S*_*q*_ ← *S*_*q*_∪{*m*});		
12 *Q* ← *Q*∪{*q*};		
13 **end**		
14 **end**		
15 **end**		
// Compute the probability of the current map cell.		
16 *P* ← 1;		
17 **for** *i* = 1 **to** ⎸*S*_*q*_⎹ **do**		
18 *m* ← *S*_*q*_(*i*);		
19 *q* ← *Q*(*i*);		
20 *P* ← *P* ⋅ (1 − *P*^*m*^(*q*));		
21 **end**		
22 *M*_*p*_(*row*, *col*) ←1 − *P*;		
23 **end**		
24 **end**		

Afterwards, the probability of the current pixel is computed from line 16 to line 22 taking advantage of the data previously stored in *S*_*q*_ and *Q*. In the case of pixels not observed by any measurements, line 22 assigns zero probability to them.

As it can be observed in the algorithm, line 7 and the check in line 8 are executed *R* ⋅ *C* ⋅ |*S*| times. As an example, in the particular experimental setup described in Section Experimental setup, the number of measurements |*S*| is 68434, including the port and the starboard sensing heads. If a map of 1000 × 1000 pixels is to be built, then the probabilistic model has to be evaluated, in the worst case, 6.8434 ⋅ 10^10^ times. Line 10 is also likely to be executed in a large number of iterations. As this may be extremely time consuming, it is crucial for these three lines to be fast.

However, the most time consuming part of the algorithm lies in the loop between lines 16 and 22 because the probabilistic model *P*^*m*^(*q*) is evaluated there. As the number of iterations in this loop depends on the measurements that are likely to observe the current pixel, having an accurate rejection criteria when building *S*_*q*_ is very important.

Finally, we would like to emphasize that it is not necessary to store the probability layer *M*_*P*_ as the values for each pixel are never re-used after the corresponding cell in the echo intensity layer *M*_*I*_ is evaluated. However, the probability layer could also be used in other tasks to let the user or other higher-level AUV functionalities know how confident it can be about the information in every mapped region.

Some experiments showing the probability layer *M*_*P*_ using the three aforedescribed SSS models with real data in a large subsea mission are provided in Section Experimental results.

### The echo intensity layer

This section describes our approach approach to build the echo intensity layer *M*_*I*_. The first step is to compute the echo intensity value *V*^*m*^(*q*) for a pixel *q* according to a measurement *m*. This is achieved by linearly interpolating the values in $I_{g}^{m}$ for each pixel corner and then computing the mean of the four obtained values as follows:

$$\begin{array}{c} V^{m}\left(q\right)=\left\{\begin{array}{c} 0,m\notin S_{q}\\ \frac{\sum_{i=0}^{3}\left(w_{1}I_{g}^{m}\left(\lceil \frac{r_{q,i}^{m}}{\delta_{g}}\rceil \right)+w_{2}I_{g}^{m}\left(\lfloor \frac{r_{q,i}^{m}}{\delta_{g}}\rfloor \right)\right)}{4},m\in S_{q} \end{array}\right. \end{array}$$ (31)

where $w_{1}=\frac{r_{q,i}^{m}}{\delta_{g}}-\left\lfloor \frac{r_{q,i}^{m}}{\delta_{g}}\right\rfloor$ and *w*_2_ = 1− *w*_1_. In case the pixel *q* is not observed by measurement *m*, we assign it the arbitrary value of 0.

Our approach to compute the echo intensity value *V*(*q*) corresponding to a pixel *q* taking into account all the measurements *m* ∈ *S*_*q*_ that may have observed it is to weight the individual *V*^*m*^(*q*) according to the corresponding probability *P*^*m*^(*q*). In this way, an echo intensity value corresponding to one measurement is more influential in the final value if the measurement has higher probability of having observed the pixel. That is:

$$\begin{array}{c} V\left(q\right)=\sum_{m\in S_{q}}P^{m}\left(q\right)V^{m}\left(q\right) \end{array}$$ (32)

By computing *V*(*q*) for all the pixels in the map, the echo intensity layer *M*_*I*_ is built. Some experiments regarding the echo intensity layer are presented in Section Experimental results.

## Geometric map building

Fig 7 shows that some pixels may not be observed according to the proposed probabilistic model. In that example, neither pixel *q*0 nor pixel *q*1 were observed and, thus, the proposed approach to build the echo intensity layer will assign them a value of zero.

However, these two pixels exemplify two different situations. As for pixel *q*0, its value should be certainly set to zero or to any value denoting there is no information about it. Contrarily, the value of pixel *q*1 could be extrapolated from the surrounding measurements. By doing so, although the probability to observe *q*1 is actually zero, the visual gaps in the resulting map can be properly filled.

**Fig 8:**
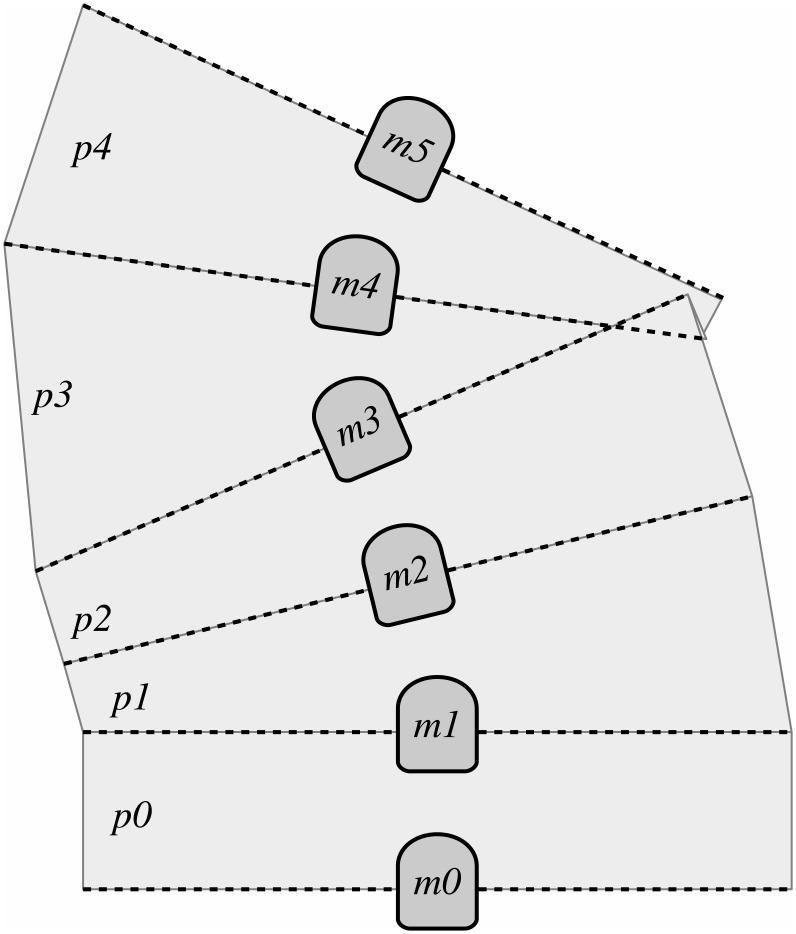
A polygonal mesh is used to perform the extrapolation. Each polygon *p*_*i*_ in the mesh is constructed by joining the acoustic axes of the measurements *m*_*i*_ and *m*_*i*+1_.

To perform the extrapolation, which is called the geometric map because it is based on pure geometry, we start by representing the environment as a polygonal mesh. The four edges of each polygon correspond to the acoustic axes of consecutively gathered measurements joined by their extrema as illustrated in Fig 8, where each polygon *p*_*i*_ is built by joining the acoustic axes of measurements *m*_*i*_ and *m*_*i*+1_.

Then, every pixel in the map is checked against all the polygons to decide whether it is inside any of them. A pixel is said to be inside a polygon if at least one of its corners is inside. If the goal of the geometric map building is only to fill the gaps of the echo intensity layer, this step can be extremely speed-up by checking only those pixels whose probability to be observed is zero.

If the pixel is inside, its four corners are projected to all of the involved acoustic axes and an echo intensity value for each of them is computed through linear interpolation. Also, the perpendicular distance from each of these corners to all of the involved acoustic axes is computed. The final value assigned to this pixel is a weighted mean of the obtained echo intensities whose weights are inversely proportional to the corresponding perpendicular distance.

By means of this method, the gaps in the echo intensity layer can be filled. Also, this approach can be used standalone to build an alternative representation of the sea floor.

## Experimental results

### Experimental setup

The experiments shown in this section have been performed using an Imagenex SportScan SSS with two sensor heads attached to an EcoMapper AUV. The main parameters of this system are shown in Table 1. As for the mean altitude being *h* = 5*m*, we would like to emphasize that the true values during all the mission are almost constant and very close to the mean value, which is a common situation in underwater surveying missions. The AUV was also endowed with a DVL, providing dead reckoning and altitude, and a GPS providing position data when in surface. Moreover, the DVL is also endowed with a compass and, thus, it provides absolute heading data.

**Table 1:** Parameters of the SSS used in this paper.

*α*	30°
*φ*	3°
*θ*	20°
*f*	800KHz
*λ*	1.95mm
*r*_*s*, *max*_	30m
Resolution *δ*_*s*_	0.12m
Bins per swath	250 port, 250 starboard
Mean altitude	5m

The experiments have been performed using an Imagenex SportScan SSS with two sensor heads attached to an EcoMapper AUV. This table shows its main parameters.

**Fig 9:**
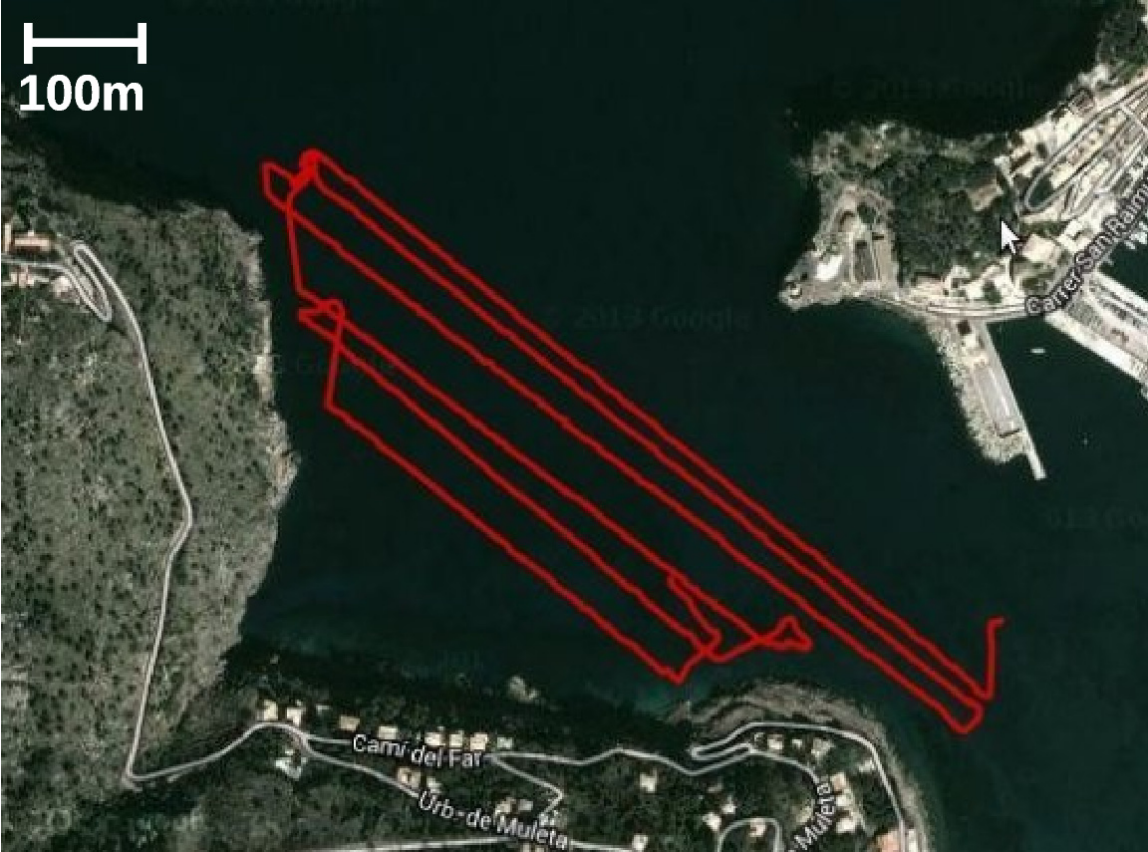
The mission. Trajectory followed by the AUV (red line) overlayed to a Google Maps view of the environment.

The AUV mission consisted of a sweeping trajectory along more than 4Km in Port de Sóller (Mallorca, Spain). During the straight transects, the AUV was underwater gathering SSS and DVL data. During the turns, the robot emerged to obtain the GPS position. Fig 9 depicts the AUV trajectory, computed using the localization approach described in Section Localization.

Port de Sóller is a small bay in the North coast of Mallorca (Balearic archipelago, Spain). The seafloor of the bay is sandy with little sea-grass meadows laid out quite regularly. Rock outcrops are frequent at the mouth of the bay. The experimental area depth ranges from 12 to 22m and the significant points appearing on the sonar images mostly correspond to stones and Posidonia Oceanica plants. Along some of the transects the sonar visualizes a 80cm diameter pipe used for fresh water supply.

All the SSS measurements used in these experiments, both raw and processed, are available in [24] together with some high-resolution maps and additional mission data.

Along the experiments, some data regarding the time consumption will be provided. In these cases the measured times correspond to a Matlab implementation running on Ubuntu 14 and an Intel i7 CPU at 3.1GHz.

### The flat floor assumption

Let us evaluate the effects of the flat floor assumption by parametrizing the error models presented in Section The flat floor assumption with the specific SSS used in this paper.

**Fig 10:**
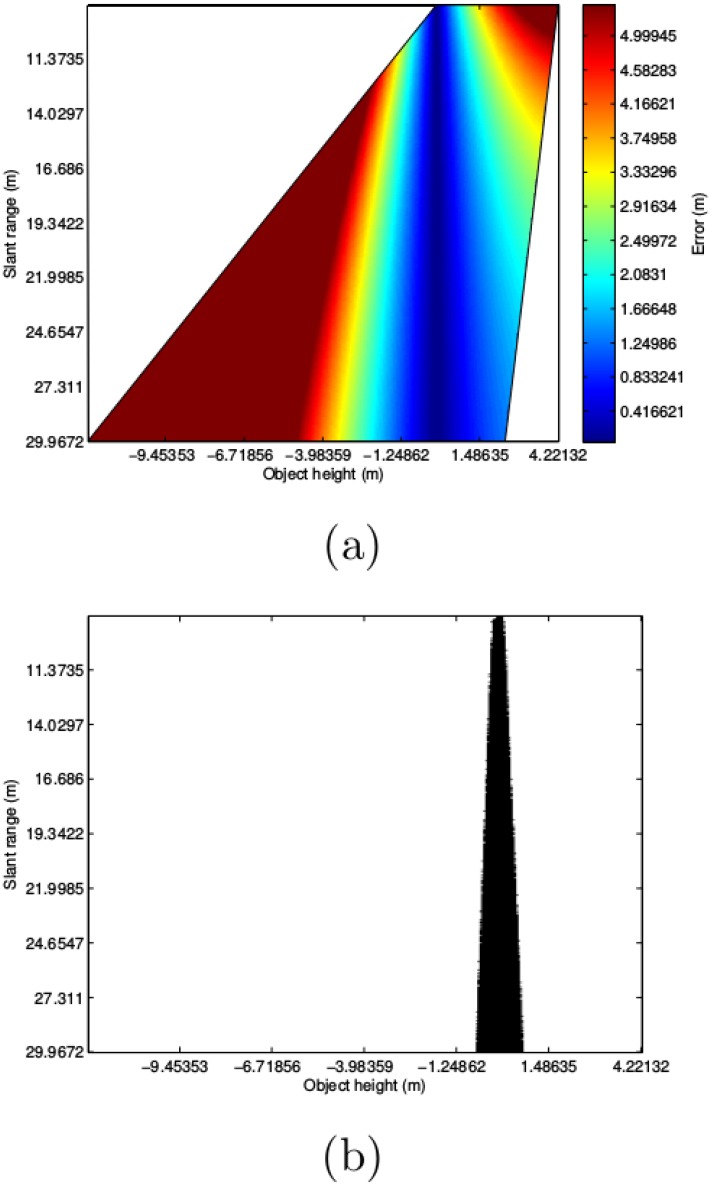
Output of the error model. (a) Error model as a function of the unknown object height and the measured slant range. The error is computed using Eq 3 and represents the difference, in meters, between the actual ground range corresponding to a detected object, and the estimated ground range if the flat floor assumption is performed. (b) Fully neglectable errors. The errors in (a) that are below the sensor resolution are depicted here and considered neglectable.

Fig 10a shows the errors according to Eq 3. For each slant range, the minimum and maximum object altitude have been computed using Eqs 4 and 5. As for the AUV altitude, values below the one shown in the figure have not been considered as in that cases it is not possible to perform the flat floor assumption. Let the smallest possible slant range according to this criteria be denoted by *r*_*s*, *min*_. The largest value *r*_*s*, *max*_ = 30*m* is one of the sensor parameters.

Fig 10b shows all the combinations of slant range and object altitude that lead to fully neglectable errors. In the worst case, which appears with small slant ranges, object altitudes between −16cm and 16cm lead to fully neglectable errors. In the best case, corresponding to large slant ranges, the flat floor assumption leads to fully neglectable errors for objects with altitudes ranging from −66cm to 71cm. If the reasonable mean slant range of 15m is considered, then the flat floor assumption leads to no errors for object altitudes between −33cm and 32cm.

To illustrate this analysis, let us focus on some particular data in the dataset used in this paper. To this end, let us first introduce one additional concept. It is well known that the height of an object observed by a SSS can be computed by measuring the length of the projected shadow. This task is extremely difficult and error prone to be automated, but for some especially contrasted regions it can be done manually. Let *r*_*s*1_ be the slant range of the last non-shadow point of the object whose height is to be computed. Let *r*_*s*2_ be the largest slant range of the shadow projected by the object. It is straightforward to see that the object height is:

$$\begin{array}{c} h_{p}=h\cdot \left(1-\frac{r_{s1}}{r_{s2}}\right) \end{array}$$ (33)

**Fig 11:**
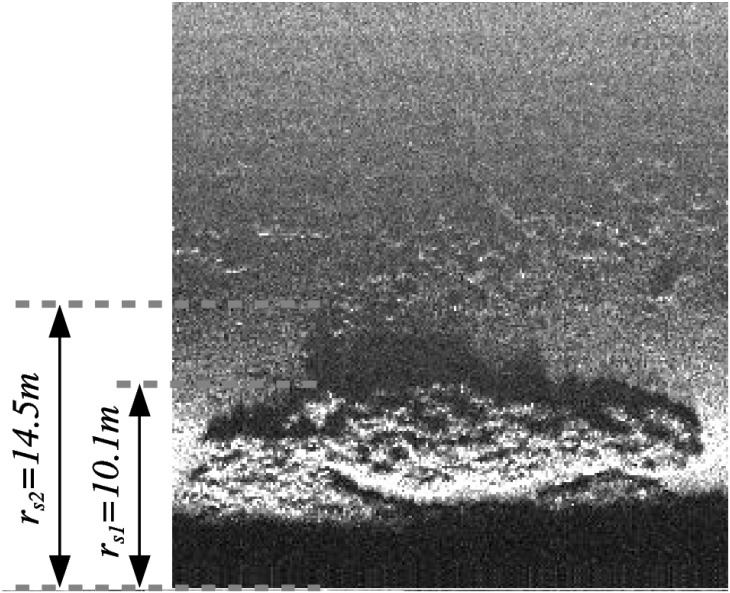
Structure with the largest shadow in our dataset. The values *r*_*s*1_ = 10.1*m* and *r*_*s*2_ = 14.5*m* have been found by manually selecting the shadow. This example is used to determine the maximum ground range error *e*_*max*_ in our dataset due to the flat floor assumption. This maximum error is 0.7m and is not fully neglectable, but it leads only to a 1.16% of misplacement with respect to the whole SSS range.

By visual inspection, we found that the structure shown in Fig 11 is the one with the largest projected shadow and, thus, the one with largest altitude. By means of Eq 33, the obtained height is 1.51m. According to Eq 3 the flat floor assumption will, in this case, lead to an error of 0.7m. Thus, the error is not fully neglectable as it is larger than the sensor resolution. However, as the error in this case is 5.8 times the sensor resolution, the highest point of the structure will be misplaced less than 6 bins in the resulting acoustic image. As the acoustic image has 500 bins, this means only a 1.16% of misplacement with respect to the whole SSS range. Let this error corresponding to the highest structure in the dataset be denoted by *e*_*max*_.

As for the effects of the across-track slope, computing Eqs 6 and 7 using our SSS parameters shows that slopes within the interval *s*_*min*_ = −2.24% and *s*_*max*_ = 2.39% lead to fully neglectable errors.

The results obtained by configuring the models with our particular sensor parameters are summarized in Table 2. This data shows that the flat floor assumption leads to small errors and, in most cases, to fully neglectable errors in the scenarios where our AUV is deployed and suggests that similar configurations and environments would lead to a similar conclusion.

**Table 2:** Flat floor assumption summary.

	*r*_*s*, *max*_	*r*_*s*, *min*_	*r*_*s*, *avg*_
$h_{p,min}^{\prime }$	−66cm	−16cm	−33cm
$h_{p,max}^{\prime }$	71cm	16cm	32cm
*s*_*min*_	−2.24%		
*s*_*max*_	2.39%		
*e*_*max*_	1.16%		

Results obtained by configuring the models related to the flat floor assumption with our particular sensor parameters.

### Swath correction

This Section provides some experimental results showing the effects of the swath correction methods presented in Section Swath correction. The sensor parameters used are those presented in Table 1, except for the altitude *h*. For the sake of clarity, regions where the AUV altitude changed have been chosen here.

**Fig 12:**
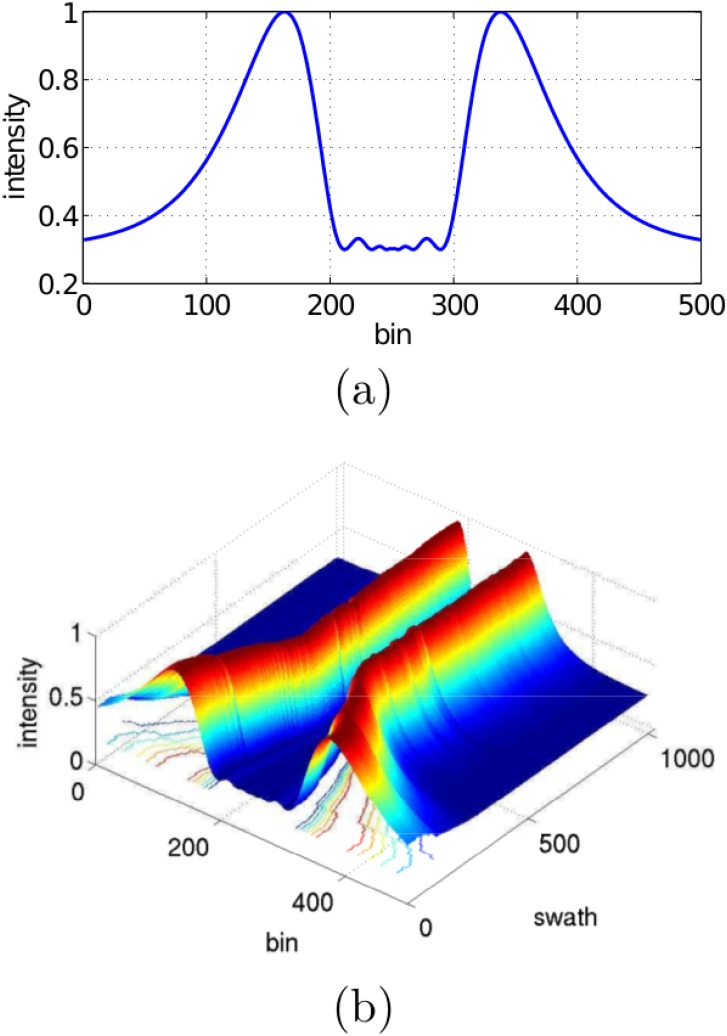
Estimated ensonification intensities according to the proposed model. (a) Intensities corresponding to a single swath involving both port and starboard sensing heads. The intensities have been normalized to a value between 0 and 1. (b) Intensities corresponding to a transect. The *swath* axis denotes consecutively gathered swaths.

Fig 12a shows an example of the ensonification intensity for a whole swath according to the model presented in Section Intensity correction parametrized for our sensor at an altitude *h* = 6.54*m*. In this case, bins denote slant ranges. It provides a fair representation of the sensor behavior, as it shows two main intensity peaks, one for each sensor head. The intensity peaks correspond to the main acoustic lobes, but they are also influenced by the distance of each bin to the sensor. Because of that, each peak is not symmetric with respect to its acoustic axis. The central region clearly reflects low ensonification intensity of the blind zone because of its proximity to the main lobe boundary.

Fig 12b shows a set of 1000 ensonification intensity curves corresponding to the the beginning of a transect. During the first 330 swaths, the vehicle altitude descended from *h* = 9.4*m* to *h* = 3.9*m*. From swath 331 onward, the vehicle altitude was almost constant and close to *h* = 3.9*m*. As the AUV descends, the two intensity peaks are getting closer and the region corresponding to the blind zone becomes smaller, stabilizing when constant altitude is reached. This is consistent with SSS imagery, in which the blind zone changes depending on the altitude.

By comparing Fig 12 and the swath examplified in Fig 3, it is clear that by removing the ensonification intensity component the resulting swath will be a much more realistic representation of the sea floor.

**Fig 13:**
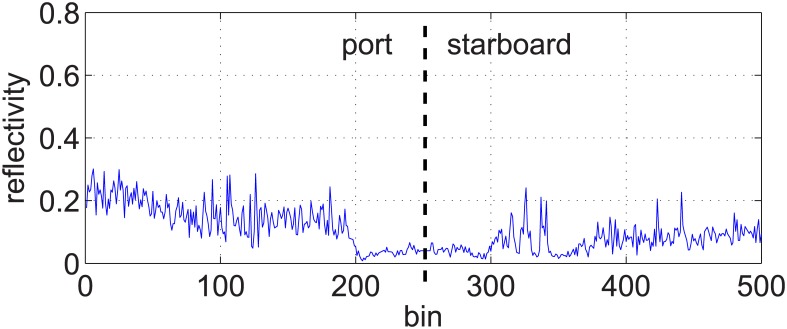
Reflectivity corresponding to a single swath. The image shows the swath in Fig 3 corrected according to the model in Eq 12.

Fig 13 shows the swath in Fig 3 corrected according to Eq 12. It can be observed how the high intensity peaks have been removed and, thus, the corrected swath mainly holds information about the sea floor.

**Fig 14:**
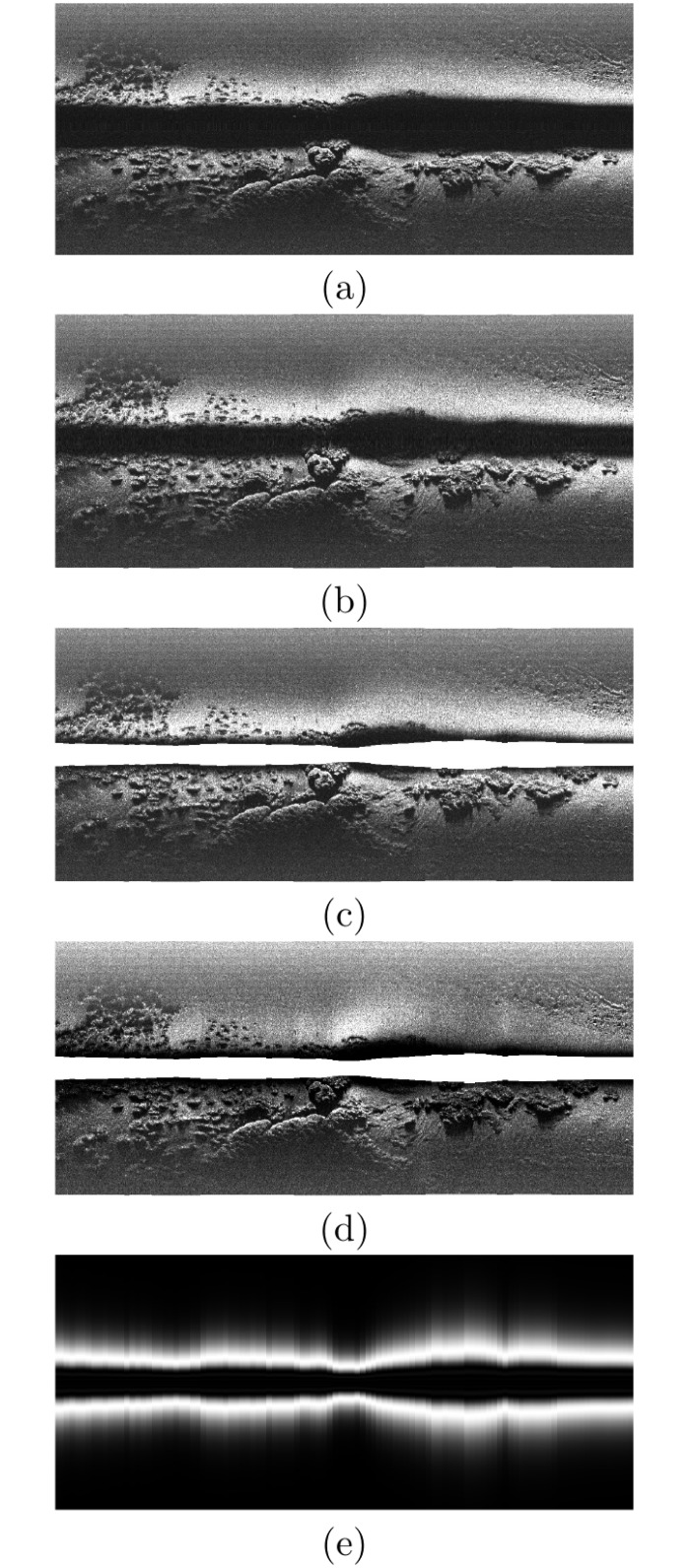
Acoustic images. The columns correspond to consecutively gathered swaths, either corrected or not. (a) Original. (b) Slant correction. (c) Slant correction + Blind zone removal. (d) Slant correction + Blind zone removal + Intensity correction. (e) Ensonification intensity model. In this case, each column corresponds to the model output for each swath.

Fig 14 summarizes the whole swath correction processes using a set of 1000 consecutively gathered swaths. The image in Fig 14a shows the raw data provided by the SSS. The effects of the uneven ensonification can be clearly appreciated above and below the blind zone. Fig 14b shows the slant corrected data as described in Section Slant correction. Fig 14c shows the effect of removing the blind zone, as described in Section Blind zone removal, to the slant corrected data. Fig 14d shows the effects of the intensity correction as described in Section Intensity correction. It can be observed how the bright regions close to the blind zone are almost completely removed. Finally, Fig 14e shows the estimated ensonification intensity according to Eq 9.

**Fig 15:**
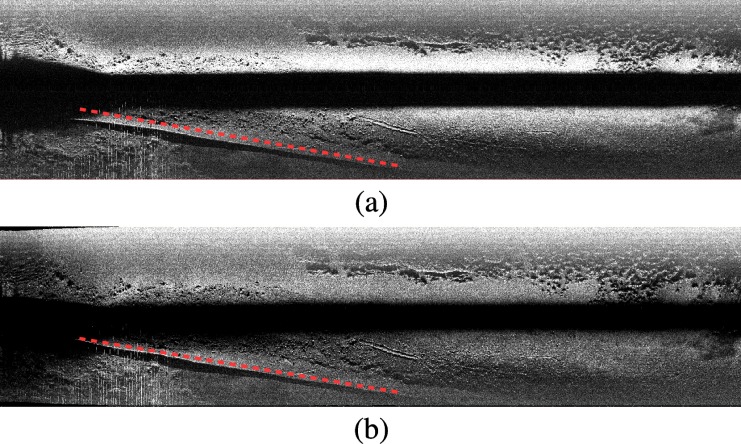
Example of the improvements due to the swath correction. (a) Raw SSS data showing a distorted straight underwater pipe. (b) Corrected data. The pipe image is corrected to its actual straight shape. The uneven brightness is also improved due to the intensity correction process. In both cases a red line is overlayed to emphasize the geometric improvement.

Fig 15 shows an example to illustrate how the proposed approach generates imagery that better captures the sea-floor geometry than the original SSS data. In particular, Fig 15a corresponds to the raw SSS measurements gathered at an area where a straight underwater pipe was observed. The pipe appears clearly distorted. Fig 15b shows the same data after applying slant and intensity correction. As a result of the former process, the pipe shape is corrected to the straight line it should be. The uneven brightness in the original image is also corrected in that case thanks to the intensity correction.

There are several tasks in which properly knowing the geometry of underwater structures is important. For example, in industrial tasks such as underwater cable or pipe inspection in which wrong geometry estimates may lead to wrong defect detections. Also, in biological surveys to estimate the growing of algae population a proper geometry reconstruction is crucial to obtain accurate measurements. Moreover, in both cases, the structures to be analyzed, either biological or industrial, are usually close to the sea floor, rendering the swath correction even more effective.

### The SSS model

This Section provides some illustrative examples of the three approaches presented in Section The SSS model to model the probability of a map cell to be observed.

**Fig 16:**
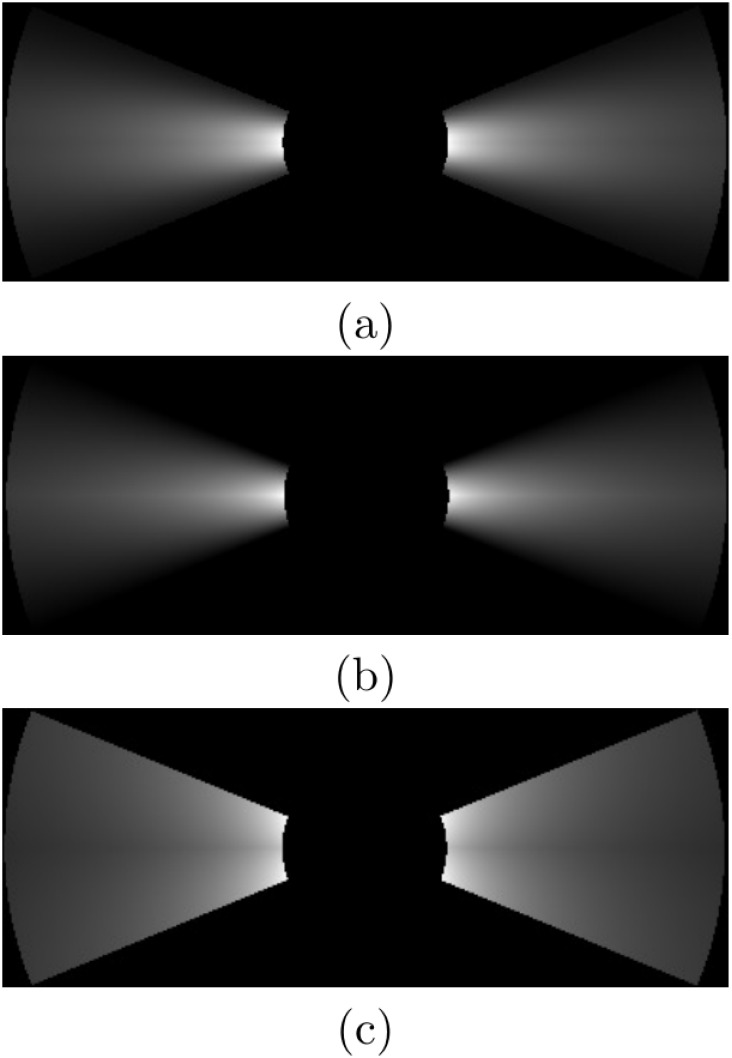
SSS probabilistic models of two sensing heads. The parameters used are *r*_*g*, *min*_ = 2.5*m*, *r*_*g*, *max*_ = 10*m*, *ϕ* = 45^*o*^ and *δ*_*M*_ = 0.05*m*. The picture shows the observation probabilities involving a single SSS measurement according to (a) Gaussian PDF ($P_{G}^{m}\left(q\right)$), (b) Triangular PDF ($P_{T}^{m}\left(q\right)$) and (c) Uniform PDF ($P_{U}^{m}\left(q\right)$). The gray levels have been re-scaled to 0–1 for the sake of clarity.

Fig 16 exemplifies (a) the Gaussian approach, (b) the Triangular approach and (c) the Uniform approach by plotting the probabilities $P_{G}^{m}\left(q\right)$, $P_{T}^{m}\left(q\right)$ and $P_{U}^{m}\left(q\right)$ for all the map cells in the image and a single SSS measurement. The SSS parameters used in this example do not correspond to the SSS used in the other experiments, and they have been chosen to provide illustrative examples.

It can be observed how the Triangular approach leads to results quite similar to those of the Gaussian approach, whilst the Uniform approach produces a very simplistic representation. In all cases the probability decreases with distance because the angular range where the pixel lies is smaller for farther pixels.

Table 3 shows the time spent in the probability computation when building the images in Fig 16. As it can be observed, the Triangular model leads to a significant reduction of execution time with respect to the Gaussian approach. Also, it is clear that, in terms of computation time, the Uniform model provides the best results.

**Table 3:** Time spent by $P_{G}^{m}\left(q\right)$, $P_{T}^{m}\left(q\right)$ and $P_{U}^{m}\left(q\right)$ to build the example images in Fig 16.

$P_{G}^{m}\left(q\right)$	$P_{T}^{m}\left(q\right)$	$P_{U}^{m}\left(q\right)$
1.75s	1.09s	0.58s

Time spent in the probability computation when building the images in Fig 16.

### The probability map

This Section shows some experimental results obtained when building the probability layer *M*_*P*_ using the approach described in Section The probability layer.

**Fig 17:**
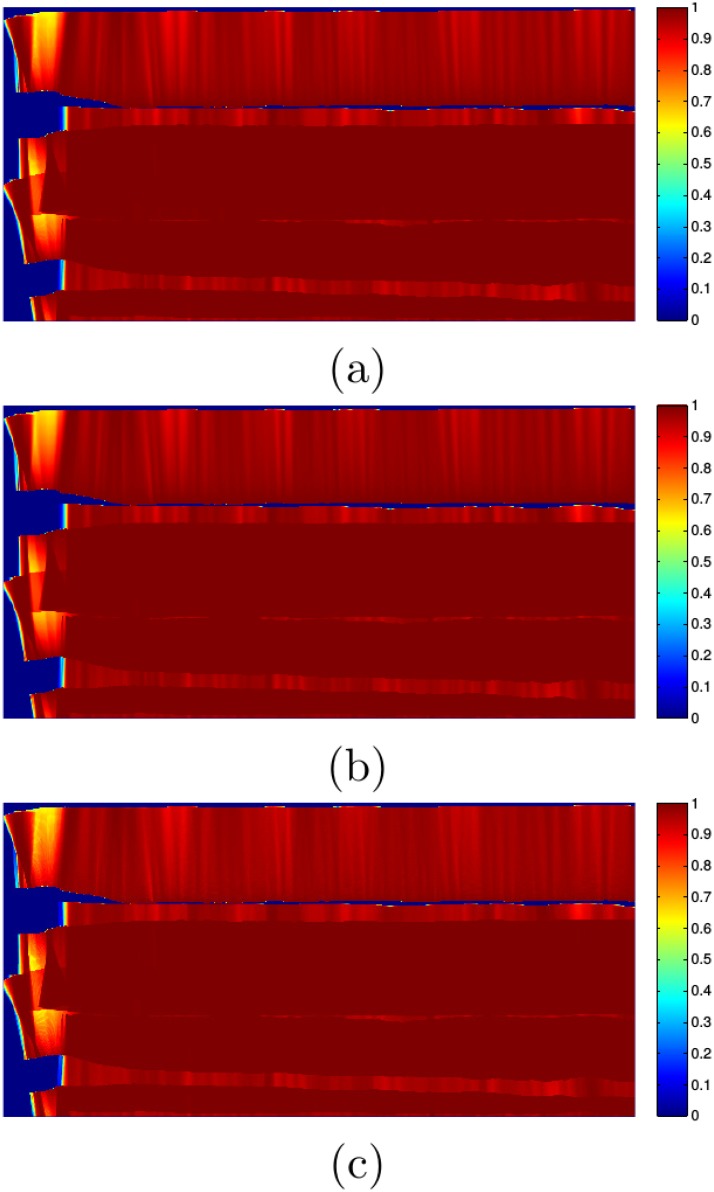
Probability layer *M*_*P*_ of a region of 180m x 90m with significant overlapping between swaths. The resolution *δ*_*M*_ is 30cm. (a) Gaussian approach $P_{G}^{m}$. (b) Triangular approach $P_{T}^{m}$. (c) Uniform approach $P_{U}^{m}$.

Fig 17 shows the probability layer *M*_*P*_ corresponding to a region of 180m x 90m observed by the SSS in the real subsea environment using (a) $P_{G}^{m}$, (b) $P_{T}^{m}$ and (c) $P_{U}^{m}$. As it can be observed, in all cases the probability is large and very close to 1 for those pixels observed by several measurements. Also, it can be observed that the three resulting probability maps are very similar. This suggests that when several readings overlap, the specific model used to compute individual probabilities has almost no influence. Also, it has to be taken into account that the large size of pixels in this case (30cm x 30cm) lead to similar values when integrating the three proposed PDFs.

**Fig 18:**
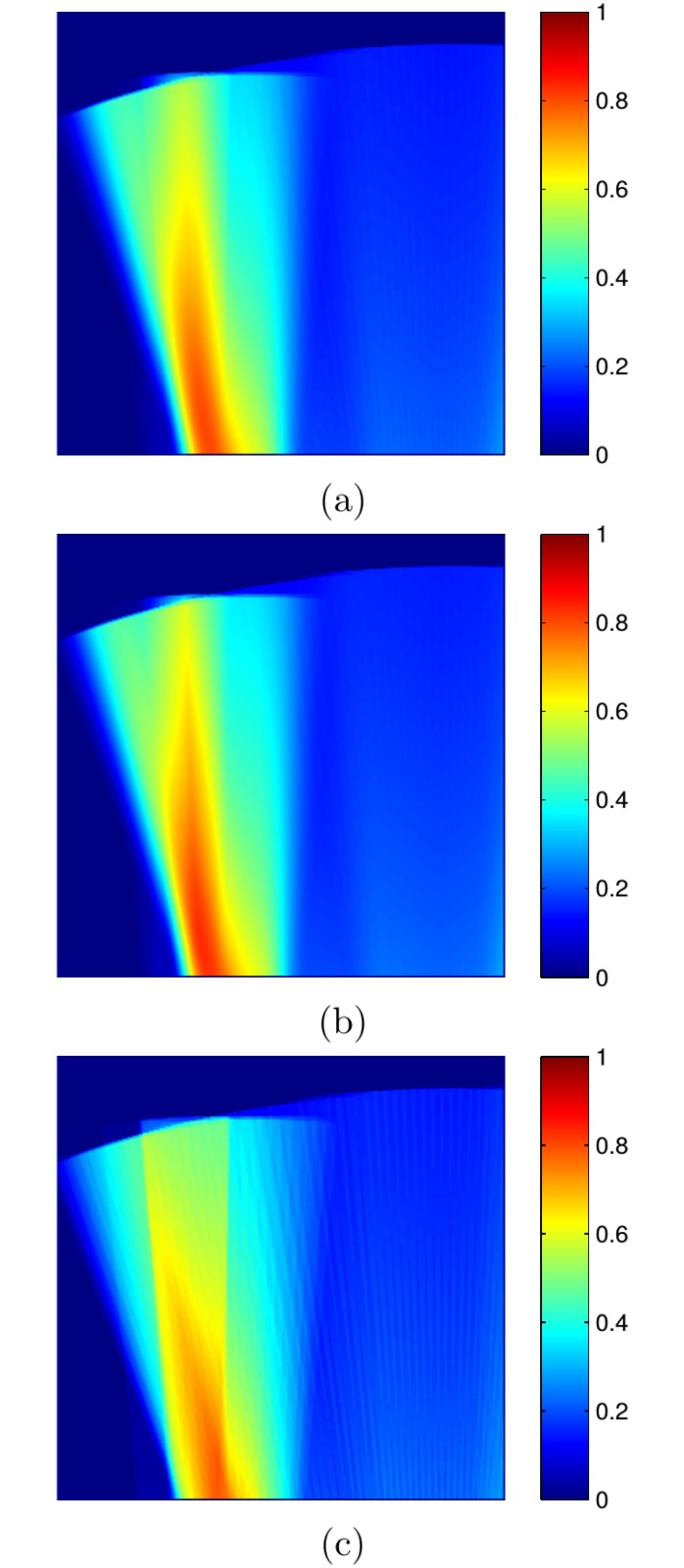
Probability layer *M*_*P*_ of region of 15m x 15m with significant overlapping between swaths. The resolution *δ*_*M*_ is 5cm. (a) Gaussian approach $P_{G}^{m}$. (b) Triangular approach $P_{T}^{m}$. (c) Uniform approach $P_{U}^{m}$.

Fig 18 shows the probability layer of a region of 15m x 15m corresponding to the upper-left corner of the images in Fig 17. The resolution is 5cm. In this case, the differences between the three models are easily appreciable. For example, changes in probability between nearby pixels are smooth in the Gaussian and the Triangular approaches, but not in the Uniform approach. Also, the probability peak close to the acoustic axis is slightly more pronounced in the Triangular approach than in the Gaussian approach.

It can be observed that the probability values are different depending on the resolution. The map can be seen as a discrete representation of a continuous probability field. Larger pixels, thus, accumulate a wider range of probabilities and, thus, have larger values. That is why the probability values are different between Figs 17 and 18.

Table 4 summarizes the time consumption when building the two previously shown groups of maps. As it can be observed, the Gaussian approach always leads to the largest computation times and the Uniform approach to the smallest ones. However, the ratio is different to the one shown in Table 3. This suggests that the time spent to evaluate the probabilistic models is very small when compared to the remaining tasks.

**Table 4:** Summary of the time consumption.

Scenario	Gaussian	Triangular	Uniform
A (Time)	29.1 min	28.81 min	28.24 min
B (Time)	5.09 min	4.76 min	4.67 min
A (Time/pixel)	0.01 s/px	0.009 s/px	0.009 s/px
B (Time/pixel)	0.0034 s/px	0.0032 s/px	0.0031 s/px

Total time and time per pixel corresponding to the maps in Fig 17 (Scenario A) and Fig 18 (Scenario B).

Also, the times spent in Scenario A and Scenario B are not proportional, as illustrated by the time per pixel, which is significantly different in both cases. As stated previously, building the map requires evaluating the probabilistic model |*S*_*q*_| times for every pixel, where *S*_*q*_ is the set of measurements in which the pixel *q* is contained. As Scenario B concentrates in a region much smaller than the one of Scenario A, a very large amount of readings can be discarded with the very simple and fast criteria of not overlapping the rectangle defined by the map. This clearly shows that having fast criteria to discard non-informative measurements is crucial in terms of time consumption.

As a conclusion, when building low resolution maps such as those in Fig 17 the specific probabilistic model barely influences the probabilistic layer. Thus, in this case the Uniform approach is a good choice as it is faster.

When building high resolution maps such as the ones in Fig 18, however, the situation changes. On the one hand, the differences between the three models arise, being the Gaussian one the preferred from this point of view. On the other hand, the influence in time consumption due to the model evaluation is larger: in the low resolution map, using the Gaussian approach leads to a 3% increase in computation time with respect to the Uniform approach, but in the high resolution map the execution time increases an 8%. Thus, from the time consumption point of view, the Uniform approach is to be considered. In these cases, deciding the model to use depends on the available computational resources and the number of map cells to compute.

### The echo intensity map

**Fig 19:**
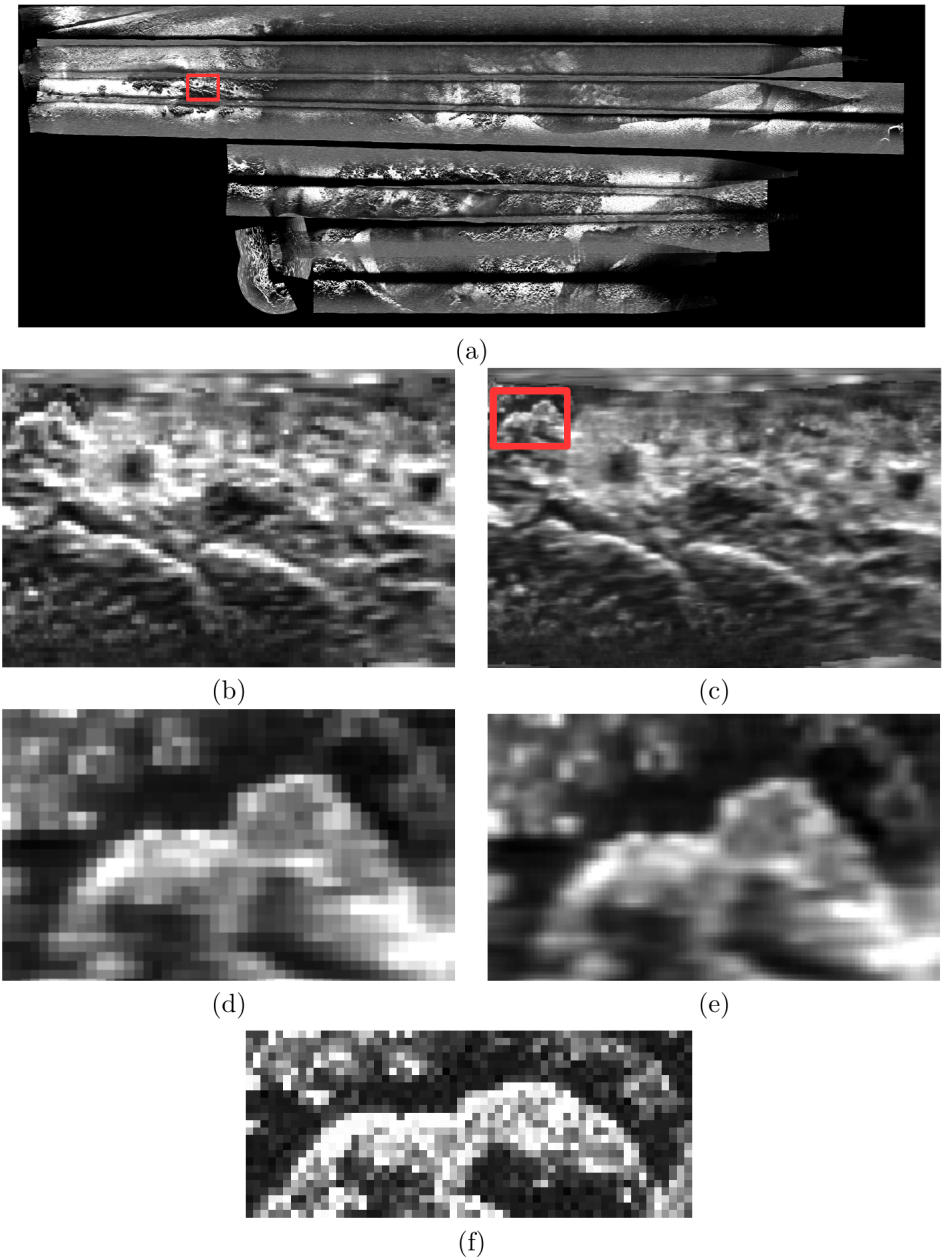
Echo intensity layer *M*_*I*_. (a) Region of 750m x 270m using a resolution *δ*_*M*_ = 30*cm*. (b) Region of 30m x 20m using a resolution *δ*_*M*_ = 30*cm* corresponding to the area inside the red rectangle in a). (c) The same region in b) using a resolution *δ*_*M*_ = 12*cm*. (d) Region of 5.7m x 3.5m using a resolution *δ*_*M*_ = 12*cm* corresponding to the area inside the red rectalngle in c). (e) The same region in d) using a resolution *δ*_*M*_ = 5*cm*. (f) Raw SSS data.

Fig 19a shows the echo intensity map built using a resolution of 30cm and corresponding to the whole mapped area. As it can be observed, the approach successfully merges the swaths when they overlap.

Fig 19b shows the region marked with a red rectangle in Fig 19a using the same resolution as before (*δ*_*M*_ = 30*cm*). The same region using a better resolution of *δ*_*M*_ = 12*cm* is shown in Fig 19c. The image quality is improved and more details can be observed. The resolution of 12cm is chosen because is the one of the SSS sonar.

Fig 19d and 19e depict the area marked with the red rectangle in Fig 19c using resolutions of 12cm and 5cm respectively, showing the ability of our proposal to achieve resolutions beyond the physical SSS limitations. The additional details that can be observed when improving the resolution are due to the combination of several overlapping measurements.

Finally, Fig 19f shows the raw SSS data that lead to the two previous images. The raw data is distorted with respect to the previous ones because it neither takes into account the actual robot motion nor the slant correction: the columns are consecutively gathered swaths and the rows are slant ranges not taking into account the changes in the AUV altitude. Thanks to all the performed processes, the image in Fig 19e makes it possible to observe the true shape of the sea bottom with finer detail than the unprocessed SSS data.

**Fig 20:**
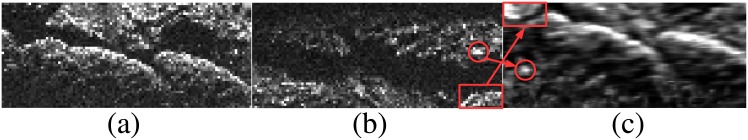
Example of data from the same sea-floor region gathered from different viewpoints. (a) Data gathered with the AUV moving south-east. (b) Data gathered with the AUV moving north-west. (c) Resulting intensity map fusing the overlapping data. The resolution is *δ*_*M*_ = 12*cm* and the mapped area is 24mx10m.

Fig 20 exempifies how the proposed map building successfully merges overlapping swaths obtained from different viewpoints. Fig 20a and Fig 20b show two sets of SSS measurements corresponding to the same area in the environment. As the AUV was moving in opposite directions when gathering each set of measurements, each image appears rotated 180° with respect to the other. Also, the objects in the sea floor were ensonified from different angles leading to a completely different imagery, although corresponding to the same area: some regions clearly visible in one of the data sets are hidden in the second one.

Both viewpoints of the same objects in the space are merged in the map shown in Fig 20c using our proposed approach, providing a more complete representation of the environment and also correcting the geometry. As it can be observed, both sets of measurements have been represented with respect to the same reference frame thanks to the localization modules. Also, the main scene details are properly fused and visible in the resulting map. For example, the bottom left region of Fig 20a contains almost no information, but that region is completed in the final map with data coming from Fig 20b. Being the data in Fig 20b rotated and having that image low echo intensities, some red markers have been added to emphasize the correspondences.

The ability of the proposed approach to fuse echo intensities makes it possible to build visually consistent maps of the environment, helping the user to have a complete representation of the observed sea floor from the partial views provided by each SSS measurement. This is especially relevant in scientific and industrial tasks in which the objects of interest may be perceived in very different ways depending on the ensonification angle. In these cases, the ability to fuse different object views into a consistent map is crucial to properly understand submerged geological or industrial structures or coral reefs, for example, where shadows may lead to very different echo intensity profiles depending on the ensonification angle.

**Fig 21:**
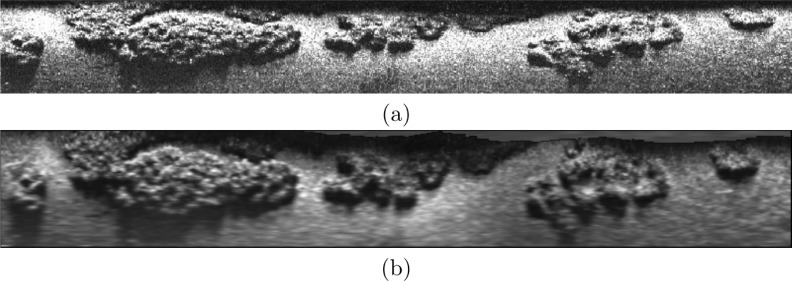
Example of the echo intensity map built using the presented approach. (a) Raw SSS data. (b) Echo intensity map *M*_*I*_ with a resolution *δ*_*M*_ = 0.09*m*.

Another example is provided in Fig 21. In this image both the raw data and the corresponding echo intensity map are shown. The improvements in resolution as well as the benefits of the echo intensity correction are clearly visible.

As for the time consumption, the results are shown in Table 5. As it can be observed, the mean time per pixel in the largest scenario is smaller than in the other ones. This suggests that, algorithmically, the time spent in operations not depending on the map size is predominant. Examples of operations not depending on the map size are discarding SSS measurements not influencing the map or computing their coordinates with respect to the global map frame. Also, the number of SSS measurements influencing the smaller regions is proportionally larger than in the large map and so is |*S*_*q*_|, thus leading to proportionally larger time consumptions in these particular smaller maps. The same explanation applies to the differences in time consumption between these results and those shown in Section The probability map.

**Table 5:** Summary of the time consumption.

Region	Resolution	Total time	Time per pixel
750m x 270m	30cm	3.78 h	0.0061 s/pix
30m x 20m	30cm	1.39 min	0.0129 s/pix
30m x 20m	12cm	8.87 min	0.0131 s/pix
5.7m x 3.5m	12cm	0.52 min	0.0124 s/pix
5.7m x 3.5m	5cm	3.07 min	0.0128 s/pix

Time consumption of the echo intensity map corresponding to the images in Fig 19.

In the two shown smaller regions, it can be observed how the time consumption increases with the resolution and the map size but the time per pixel is similar. This shows that, in similar regions where |*S*_*q*_| is likely to be similar, the mean time per pixel barely changes.

### Geometric map building

**Fig 22:**
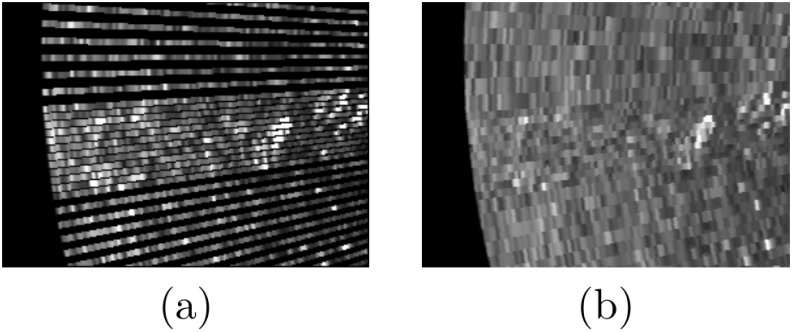
Some cells may remain unobserved due to the AUV motion. This situation corresponds to the pixel *q*1 in Fig 7. (a) Visual gaps due to unobserved map cells. (b) Gaps filled with the Geometric map approach.

Fig 22a exemplifies a situation where some gaps appear when building the echo intensity map. Whereas pixels outside the maximum range should be left to zero, the value of those map cells between two measurements could be extrapolated to fill the visual gaps by means of the Geometric approach described in Section Geometric map building.

We would like to emphasize that these gaps would be rarely observable under our particular sensor configuration. For example, in order to build the image in Fig 22a a specific region where the robot was performing a turn was selected and mapped using a resolution of 5cm. Also, for the gaps to be clearly visible, the opening *ϕ* was set to 0.5°, six times below the actual one. Using other sensors with lower measurement rate, the gaps may appear more frequently.

Fig 22b shows the same image where the gaps have been filled according to the geometric approach. As it can be observed, there is a continuity in most cases between the echo intensity map and the values assigned to gaps by the geometric approach.

**Fig 23:**
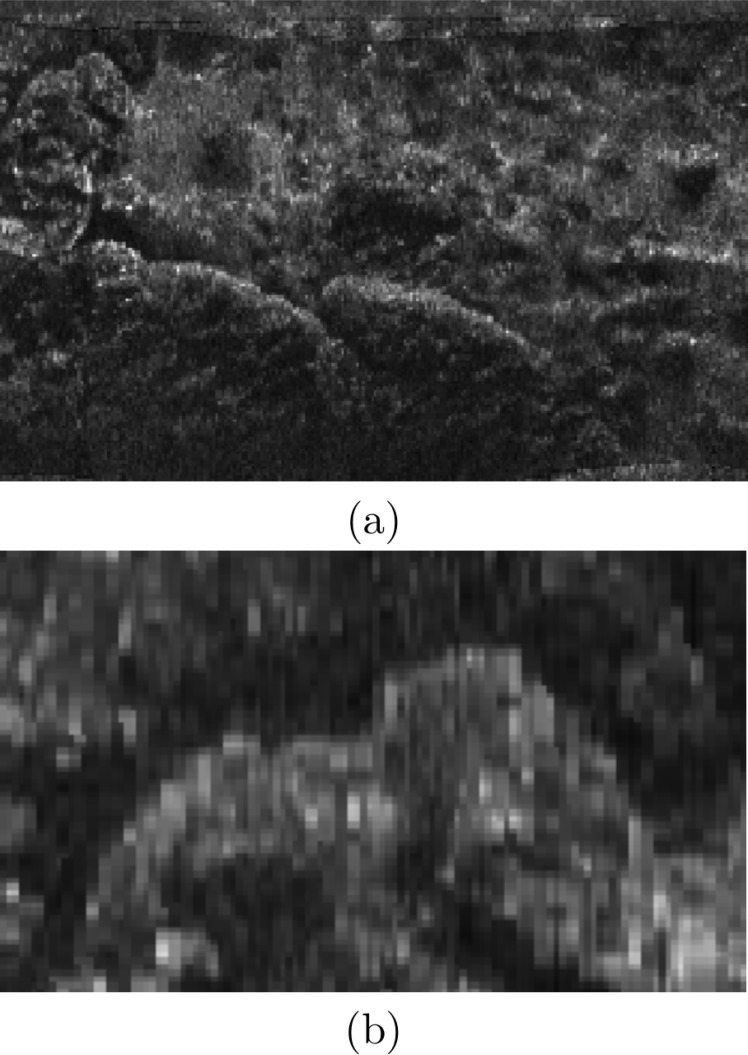
The geometric approach can also be used standalone to build the maps. This figure shows two examples build with (a) a resolution *δ*_*M*_ = 12*cm* and (b) a resolution *δ*_*M*_ = 5*cm*.

Fig 23 provides two examples showing that the geometric approach can be used standalone to build maps. Fig 23a and 23b correspond to the same regions and resolutions of Fig 19c and 19e. As it can be observed, the resulting maps clearly depict the environment. However, they are significantly influenced by the SSS noise and almost no improvements can be observed when increasing the resolution beyond the one of the SSS.

As for the time consumption, building these two geometric maps spent 4.81 min and 1.16 min, far below the time required to build the echo intensity map corresponding to the same regions (8.87 min and 3.07 min, as shown in Table 5).

## Conclusion and Future Work

Three different models to built the probabilistic layer have been experimentally tested. Among them, the Gaussian approach is the best in terms of model accuracy whilst the Uniform one surpasses the other two when considering the computation time. Also, it has been shown that for low resolution maps, the three models lead to similar results. Accordingly, it is reasonable to use the Gaussian model for high resolution maps (i.e. resolutions below the SSS one) and the Uniform or the Triangular otherwise.

The echo intensity map approach has shown to be able to provide accurate maps at better resolutions than those of the SSS by combining overlapping measurements. However, if SSS with low measurement rates are used, some gaps may appear in the map. To avoid this problem, the Geometric approach has been introduced as a method to fill such gaps.

The probabilistic layer *M*_*P*_ and the intensity layer *M*_*I*_ can be used together to provide the user or the navigation modules with information not only about the sea floor structure but also about how good is that information. This could be used, for example, to decide which areas have to be re-visited in order to reinforce the available information.

**Fig 24:**
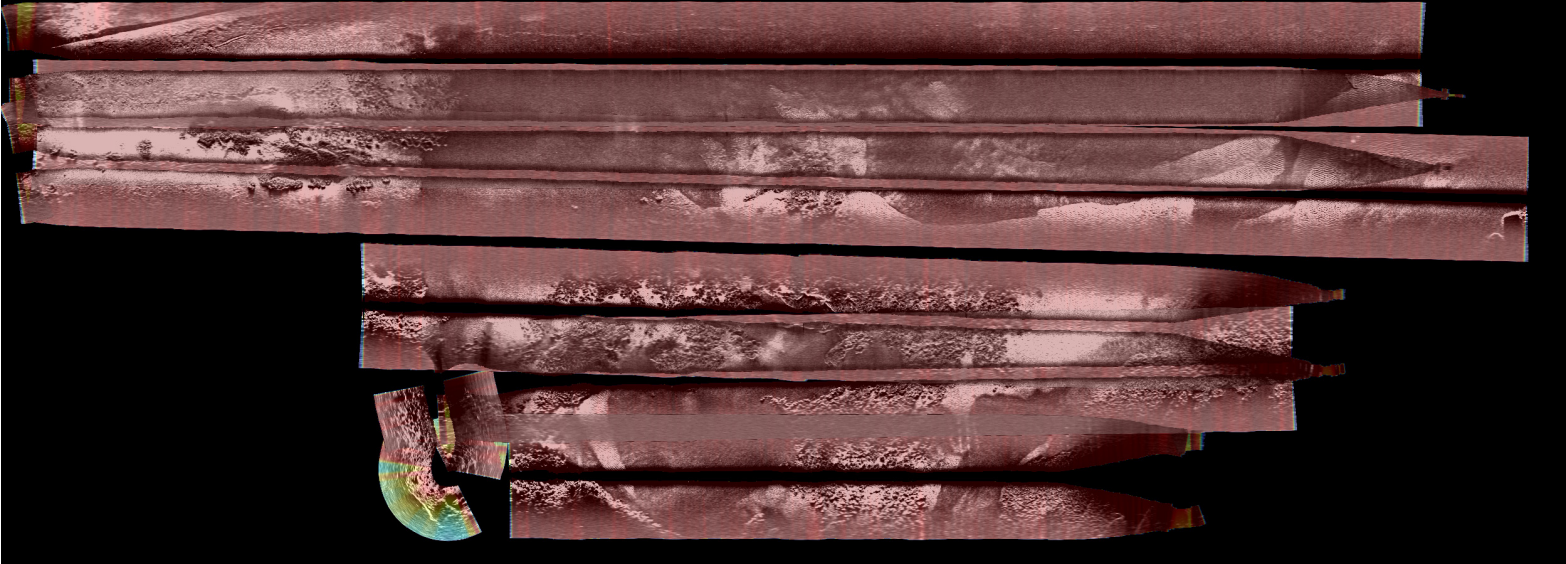
Probabilistic and intensity maps overlayed. The probabilistic shows the probability of each cell to be observed, ranging from blue (low probability) to red (high probability). The echo intensity layer shows the estimated sea floor structure.

Fig 24 summarizes this idea. This figure shows the probabilistic map, built using the Gaussian approach, and the echo intensity map of the whole explored area in our experimental setup (750m x 270m) using a resolution *δ*_*M*_ = 30*cm*. In this image, red areas denote regions with high probability of being properly represented in the map and blue areas regions with lower probability.

As for future work, our research is now focused on three key points. First, to improve the presented models to deal with different observation angles. This is important as, depending on the observation angle, the projected shadows change. The second key point is to use the results of this study to perform underwater SLAM using SSS. This is a crucial task with very few related studies providing satisfactory results. Additionally, we are focusing on purely algorithmic aspects in order to reduce the time consumption of the presented approaches.
